# Traditional and systems biology based drug discovery for the rare tumor syndrome neurofibromatosis type 2

**DOI:** 10.1371/journal.pone.0197350

**Published:** 2018-06-13

**Authors:** The Synodos for NF2 Consortium, Robert Allaway, Steve P. Angus, Roberta L. Beauchamp, Jaishri O. Blakeley, Marga Bott, Sarah S. Burns, Annemarie Carlstedt, Long-Sheng Chang, Xin Chen, D. Wade Clapp, Patrick A. Desouza, Serkan Erdin, Cristina Fernandez-Valle, Justin Guinney, James F. Gusella, Stephen J. Haggarty, Gary L. Johnson, Salvatore La Rosa, Helen Morrison, Alejandra M. Petrilli, Scott R. Plotkin, Abhishek Pratap, Vijaya Ramesh, Noah Sciaky, Anat Stemmer-Rachamimov, Tim J. Stuhlmiller, Michael E. Talkowski, D. Bradley Welling, Charles W. Yates, Jon S. Zawistowski, Wen-Ning Zhao

**Affiliations:** 1 Children’s Tumor Foundation, New York, NY, United States of America; 2 Sage Bionetworks, Seattle, WA, United States of America; 3 University of North Carolina School of Medicine, Chapel Hill, NC, United States of America; 4 Massachusetts General Hospital and Harvard Medical School, Boston, MA, United States of America; 5 Johns Hopkins University School of Medicine, Baltimore, MD, United States of America; 6 Burnett School of Biomedical Sciences, College of Medicine, University of Central Florida, Lake Nona-Orlando, FL, United States of America; 7 Center for Childhood Cancer and Blood Diseases, The Research Institute at Nationwide Children's Hospital and Department of Pediatrics, The Ohio State University College of Medicine, Columbus, OH, United States of America; 8 Leibniz-Institute on Aging–Fritz-Lipmann Institute (FLI), Jena, Germany; 9 Indiana University, School of Medicine, Indianapolis, IN, United States of America; 10 Department of Biomedical Informatics and Medical Education, University of Washington, Seattle, WA, United States of America; 11 Department of Otolaryngology, Massachusetts Eye and Ear Infirmary, Massachusetts General Hospital and Harvard University, Boston, MA, United States of America; Columbia University, UNITED STATES

## Abstract

Neurofibromatosis 2 (NF2) is a rare tumor suppressor syndrome that manifests with multiple schwannomas and meningiomas. There are no effective drug therapies for these benign tumors and conventional therapies have limited efficacy. Various model systems have been created and several drug targets have been implicated in *NF2*-driven tumorigenesis based on known effects of the absence of merlin, the product of the *NF2* gene. We tested priority compounds based on known biology with traditional dose-concentration studies in meningioma and schwann cell systems. Concurrently, we studied functional kinome and gene expression in these cells pre- and post-treatment to determine merlin deficient molecular phenotypes. Cell viability results showed that three agents (GSK2126458, Panobinostat, CUDC-907) had the greatest activity across schwannoma and meningioma cell systems, but merlin status did not significantly influence response. *In vivo*, drug effect was tumor specific with meningioma, but not schwannoma, showing response to GSK2126458 and Panobinostat. In culture, changes in both the transcriptome and kinome in response to treatment clustered predominantly based on tumor type. However, there were differences in both gene expression and functional kinome at baseline between meningioma and schwannoma cell systems that may form the basis for future selective therapies. This work has created an openly accessible resource (www.synapse.org/SynodosNF2) of fully characterized isogenic schwannoma and meningioma cell systems as well as a rich data source of kinome and transcriptome data from these assay systems before and after treatment that enables single and combination drug discovery based on molecular phenotype.

## Introduction

Neurofibromatosis 2 (NF2) is a rare neurogenetic disorder (affecting roughly 1 in 33,000 people around the world) that is characterized by multiple schwannomas and meningiomas [1]. These are benign histologically, but their multiplicity, involving multiple cranial nerves, spinal nerve roots and peripheral nerves in people with NF2 results in severe cumulative morbidity and ultimately, death. Although bilateral vestibular schwannomas are pathognomonic for NF2, meningiomas are the second most common tumor type in NF2 and their presence is associated with increased mortality [2]. Importantly, meningiomas are the most common brain tumor worldwide and many sporadic meningiomas have somatic *NF2* mutations [3,4]. In most non-NF2 patients, meningiomas and schwannomas are effectively treated with surgery or radiation therapy. In the setting of NF2, surgery and radiation therapy are associated with reduced efficacy and increased toxicity and there are no effective drug therapies for these tumors [5–7]. Hence, effective treatments for NF2-associated schwannomas and meningiomas are a major unmet medical need for both the broad population with sporadic forms of these tumors as well as for people with the rare syndrome of NF2.

A conventional approach to developing drug therapies for tumors is for individual laboratories to test new or repurposed drugs in various *in vitro* and *in vivo* assay model systems based on hypotheses about tumor pathogenesis [8]. NF2-associated meningiomas and schwannomas result from a classic tumor suppressor gene mechanism involving inheritance of an inactivating mutation in the *NF2* gene on chromosome 22q, followed by somatic inactivation of the remaining copy of *NF2* (either by mutation or loss of a large section of the surrounding chromosome). Merlin is the *NF2* tumor suppressor protein, and loss of merlin function results in dysregulation of multiple aspects of cellular behavior [9]. This knowledge has supported the generation of *NF2/Nf2* mutant *in vivo* and *in vitro* model systems [10–20]. These are powerful tools to understand the molecular basis of tumorigenesis and to assess drug effects in a system reflecting merlin loss. However, there are relatively few assay systems available and each has advantages and limitations to consider when making a commitment to clinical translation. Additional systematic limitations in developing effective therapies for *NF2* mutant tumors include: variable metrics of efficacy applied across individual laboratories and systems, limited focus on drug targets implicated by merlin loss, and histologically specific drug assessment (meningioma versus schwannoma) rather than assessment of the effect of the underlying *NF2* mutation on drug response. To address these limitations a multi-disciplinary team created a panel of *in vitro* assay systems (representing meningioma and schwannoma, merlin wildtype and merlin-deficient, and human and murine cells) to perform traditional drug screening studies in a systematic fashion with an initial set of drugs chosen for their potential relevance to *NF2* biology.

**Fig 1:**
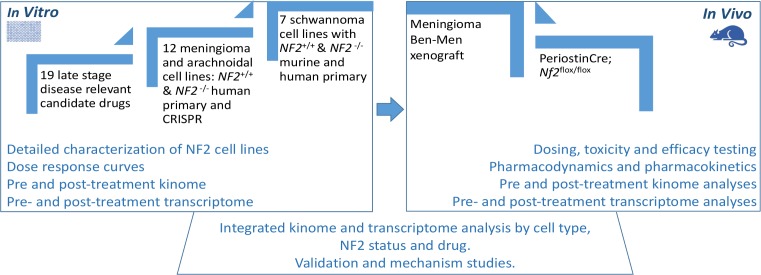
Schematic diagram for drug testing with mechanistically based candidate drugs in a traditional *in vitro* to *in vivo* pipeline and simultaneous integrated transcriptome and kinome analysis of cells and murine tumors.

Simultaneously, we performed an integrated analysis of transcriptome and kinome data across these cell culture systems at baseline and after treatment to discover tumor, species and merlin specific therapeutic targets, identify differential responses to treatment, and potentially identify mechanisms of acquired resistance. The goal was to compare drug efficacy readouts with traditional drug discovery approaches versus systems biology approaches via systematic drug assays in fully characterized isogenic pairs of schwannoma and meningioma model systems (Fig 1). The ultimate goal of this work was to create an assay system and data resource for the scientific community, with the long term goal of improving the pipeline that will identify biologically relevant therapies to be advanced for clinical development to help people with NF2 and *NF2* driven tumors.

## Results

### Development of disease-specific cell lines for drug screening

We assembled a panel of tumor-relevant cells (Schwann cells for schwannoma and arachnoidal cells for meningioma) confirmed to have suppressed or inactivated merlin and, whenever possible, their merlin-wildtype control (Table 1).

**Table 1:** Meningioma, schwannoma, arachnoidal, and Schwann cell lines used for Synodos drug screens.

Cell Line	Organism	Type	Grade	Merlin Status
Syn1	Human	Arachnoid cells (*NF2*+ CRISPR), isogenic	n/a	Wildtype
Syn2	Human	Arachnoid cells (*NF2*+ CRISPR), isogenic	n/a	Wildtype
Syn3	Human	Arachnoid cells (*NF2*- CRISPR), isogenic	n/a	Deficient
Syn4	Human	Arachnoid cells (*NF2*- CRISPR), isogenic	n/a	Deficient
Syn5	Human	Arachnoid cells (*NF2*- CRISPR), isogenic	n/a	Deficient
Syn6	Human	BenMen1, immortalized meningioma	n/a	Deficient
Syn7	Human	primary meningioma cell line	I	Deficient
Syn8	Human	primary meningioma cell line	I	Deficient
Syn9	Human	primary meningioma cell line	I	Deficient
Syn10	Human	primary meningioma cell line	I	Deficient
Syn11	Human	primary meningioma cell line	II	Deficient
Syn12	Human	primary meningioma cell line	II	Deficient
HS01	Human	Schwann cell *NF2* shRNA 45	n/a	Deficient
HS11	Human	Fetal Schwann cell	n/a	Wildtype
MS01	Mouse	Schwann cell *Nf2*^*ex2-/-*^	n/a	Deficient
MS02	Mouse	schwannoma cell *Nf2*^*ex2-/-*^	n/a	Deficient
MS03	Mouse	Schwann cell *Nf2*^*ex2-/-*^	n/a	Deficient
MS11	Mouse	Schwann cell	n/a	Wildtype
MS12	Mouse	mouse Schwann cell *Nf2*^*fl2/fl2*^	n/a	Wildtype

To represent the biology of meningiomas we assembled a primary screening cohort of 12 human cell lines, either merlin-wildtype or merlin-null. These included 5 isogenic clonally-derived arachnoidal cell (AC-CRISPR) lines [11], of which two are merlin-wildtype controls (Syn1 and Syn2) and three are merlin-deficient lines generated by CRISPR/Cas9 *NF2* inactivation (Syn3, Syn4, and Syn5), along with the immortalized merlin-deficient benign meningioma (MN) line Ben-Men-1 (Syn6) [12], as well as six patient-derived primary merlin-deficient MN lines (Syn7-Syn12). Similar growth rates were observed among the isogenic clonally-derived AC-CRISPR lines (Syn1-Syn5) and Ben-Men-1 (Syn6) line. Likewise, all chosen primary MN lines (Syn7-12) exhibited comparable growth rates.

To represent the biology of schwannoma formation, we employed a matched pair of human fetal Schwann cell (SC) lines, (HS11, wild-type) and (HS01, *NF2*-shRNA suppressed with ~3% merlin protein expression by Western blotting and ~7% RNA expression by RNA sequencing versus wild-type) as well as a series of four mouse merlin-wildtype and -deficient SC and a merlin-deficient schwannoma lineS [13,14] All control AC and SC lines expressed a merlin band at ~70 kDa by Western blot, whereas human and mouse cell lines with *NF2* or *Nf2* inactivation, respectively, failed to exhibit merlin protein (S1 Fig).

### *In vitro* meningioma and schwannoma cell culture screening with targeted compounds

Merlin is a ubiquitous protein that has both intracellular and extracellular effects [9,21,22]. Loss of functional merlin contributes to activation of multiple oncogenic pathways including Ras, Rac, PI3K and mTOR signaling pathways [11,14–16,23,24]. In addition, merlin deficiency has been linked to increased expression of several growth factor receptors including PDGFR, the EGFR family, and possibly (via Tie2) VEGFR expression [13,21,25–28].

Based on what is known about the function of merlin pre-clinically and the performance of various agents in prior preclinical and clinical evaluations, we assembled a panel of 19 drugs for initial testing in the cell culture panel that met the following requirements: (1) biological activity in clinical trials in NF2 patients, (2) activity against targets in key pathways regulated by merlin, (3) FDA-approved or in the late stages of clinical development (Table 2).

**Table 2:** FDA approved or late–stage drugs selected for cell culture screening.

Compound	Targets^a^	Inclusion Criteria^b^
LY2157299	TGF-beta/Smad	2,3[29]
Ganetespib (STA-9090)	HSP (e.g HSP90)	2,3[30]
Panobinostat (LBH589)	HDAC	2,3[27,31]
Vorinostat (SAHA, MK0683)	Autophagy, HDAC	2,3[27,31]
AR-42	HDAC	1,2,3 [27,31]
Lapatinib (GW-572016) Ditosylate	HER2, EGFR	1,2,3[24,32]
Axitinib	VEGFR, PDGFR, c-Kit	1,2,3[21,25,28,33]
AZD2014	mTOR	1,2,3[11,15,23,34,35]
OSU-03012 (AR-12)	PDK-1	2[21,36]
Perifosine (KRX-0401)	AKT	2,3 [14,36–38]
Everolimus (RAD001)	mTOR	1,2,3[11,15,23,34,35]
GSK2126458 (GSK458)	PI3K, mTOR	2,3[15,36]
GDC-0980 (RG7422)	PI3K, mTOR	2,3 [15,36]
CUDC-907	PI3K, HDAC	2,3 [13,16,36,39]
GDC-0941	PI3K	2,3[16,36]
Selumetinib (AZD6244)	MEK	2,3[24]
Trametinib (GSK1120212)	MEK	2,3[24]
Vismodegib (GDC-0449)	Hedgehog/Smoothened	2,3[38,40,41]
Bortezomib (PS-341)	Proteasome	2,3[42]

^a^TGF-beta, transforming growth factor beta; HSP, heat shock protein; HDAC, histone deacetylase; HER2, human epidermal growth factor receptor 2; EGFR, epidermal growth factor receptor; VEGFR, vascular endothelial growth factor receptor; PDGFR, platelet-derived growth factor receptor; c-Kit, tyrosine-protein kinase Kit; mTOR, mammalian target of rapamycin; PDK-1, pyruvate dehydrogenase kinase 1; AKT, protein kinase B; PI3K, phosphoinositide 3-kinase; MEK, MAPK/ERK kinase.

^b^(1) biological activity in clinical trials in NF2 patients, (2) activity against targets in a key pathway regulated by merlin, (3) FDA-approved or in the late stages of clinical development.

**Fig 2:**
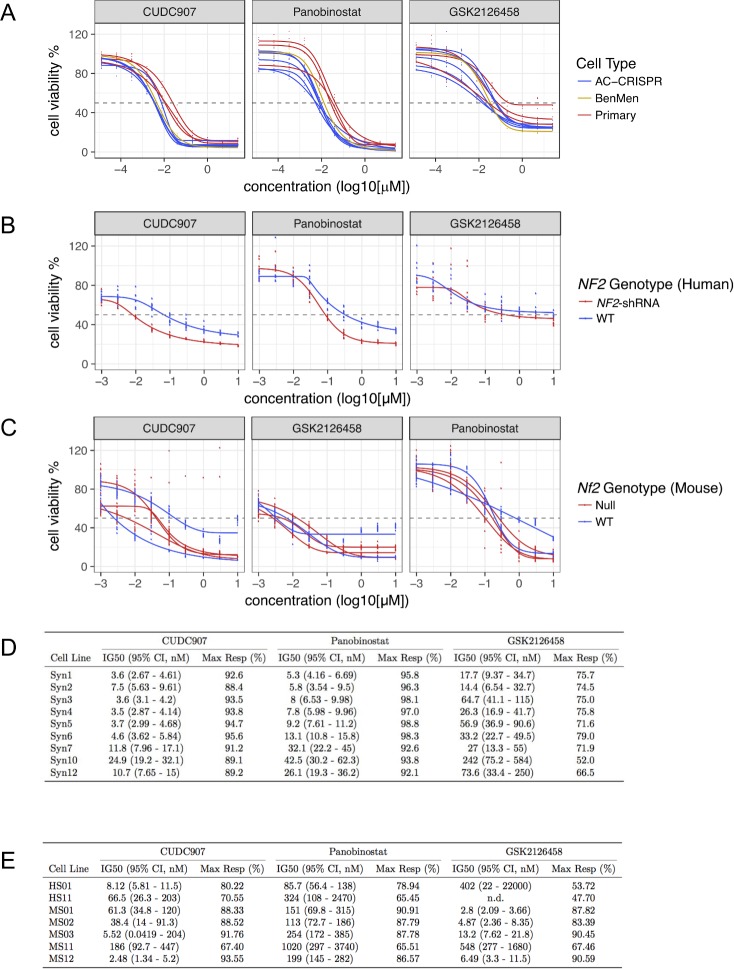
Treatment response of merlin-wildtype and merlin-deficient cells with compounds meeting efficacy metrics. **(A)** 72-hour dose response curves (DRCs) were generated for the human meningioma-relevant cell panel including all AC-CRISPR (blue), immortalized Ben-Men1 (Syn6, yellow), and 3 primary MN lines (red). (**B)** 48-hour DRCs for human SC including merlin-deficient (red) and merlin-wildtype (WT, blue) lines are shown. **(C)** DRCs generated for mouse SC/schwannoma-related lines including merlin-deficient (red) and merlin-wildtype (WT, blue) are also shown. All DRCs are expressed as percent cell viability (cell viability %) relative to vehicle treatment. Drug concentrations are expressed as log10 (μM) scale. **(D)** Dose-response metrics in AC, MN and **(E)** SC/schwannoma lines of compounds meeting efficacy metrics.

All 19 compounds were assessed in AC/MN and SC/schwannoma lines simultaneously. Dose response curves (DRCs) were generated for all meningioma-relevant (Syn1–Syn12) and schwannoma-relevant (HS01, HS11, MS01-MS12) cell lines using the CellTiter-Glo or CellTiter-Fluor viability assay (Promega), respectively. For AC/MN cells, there were two stages of screening. In the first stage, CellTiter-Glo was assessed at 72 hours of treatment using 5 dilution points (5-fold serial dilutions) of each drug, ranging from 0.04 μM to 25 μM. Based upon DRCs generated for each cell line and compound, 8 of the 19 compounds were eliminated due to minimal or no effect, even at the highest concentrations. For the remaining 11 compounds, drug treatment was expanded to a second stage. In the second stage, immortalized AC-CRISPR and Ben-Men-1 lines (Syn1-Syn6) as well as three primary MN lines (Syn7, Syn10, and Syn12) were evaluated by generating DRCs using 10 dilution points (5-fold serial dilutions) of each drug ranging from 12.8 pM to 25 μM (Fig 2A and S2A Fig). Four drugs including Panobinostat, GSK2126458, CUDC-907 and Bortezomib revealed an IG50 < 250 nM with a maximum response > 50% inhibition in all cell lines tested (Fig 2A and 2D). Of the remaining seven drugs, three (AR42, AZD2014 and GDC0980) showed an IG50 < 2 μM in all lines tested and four (Axitinib, Ganetespib, GDC0941 and Vorinostat) showed IG50 > 3 μM or no response in the primary MN lines (S1 Table). Some drugs, specifically CUDC907 in the human and mouse SC lines, as well as GSK2126458 in the mouse SC cell lines, did not reach 100% viability, indicating that these molecules have an effect at and possibly below the lowest tested drug concentration.

For the human SC panel, cell viability was assessed with CellTiter-Fluor at 48 hours with increasing concentrations at half-log concentrations, ranging from 0.001 μM to 10 μM. Five drugs, including Panobinostat, GSK2126458, CUDC-907, Bortezomib and Ganetespib, revealed IG50 <500 nM with a maximum response > 50% inhibition in merlin-deficient HS01 (Fig 2B and 2E, S2B Fig). All other drugs showed IG50 > 2 μM or not determined (n.d.; maximum response <50%) (S2B Fig and S2 Table). Mouse SC lines treated with Panobinostat, GSK2126458, CUDC-907 or Bortezomib revealed IG50 of ≤ 1 μM with a maximum response > 55% inhibition in all merlin-deficient cell lines tested (MS01, MS02 and MS03) (Fig 2C and 2E, S2 Table). All other drugs tested showed variable IG50 ranging from 2 nM to > 5 μM or could not be determined in merlin-deficient mouse SC or schwannoma lines due to DRC that could not be fitted (S3 Fig and S2 Table).

Comprehensive evaluation of the compounds’ performance across both AC/MN and SC/schwannoma assay systems *in vitro* with the goal of selecting drugs that had the highest likelihood of efficacy against both tumor types *in vivo* resulted in three compounds being selected to be advanced to *in vivo* testing: CUDC-907 (dual PI3K/HDAC inhibitor), Panobinostat (HDAC inhibitor), and GSK2126458 (dual PI3K/mTOR inhibitor).

### Variables associated with drug response in meningioma and schwannoma cell culture systems

**Fig 3:**
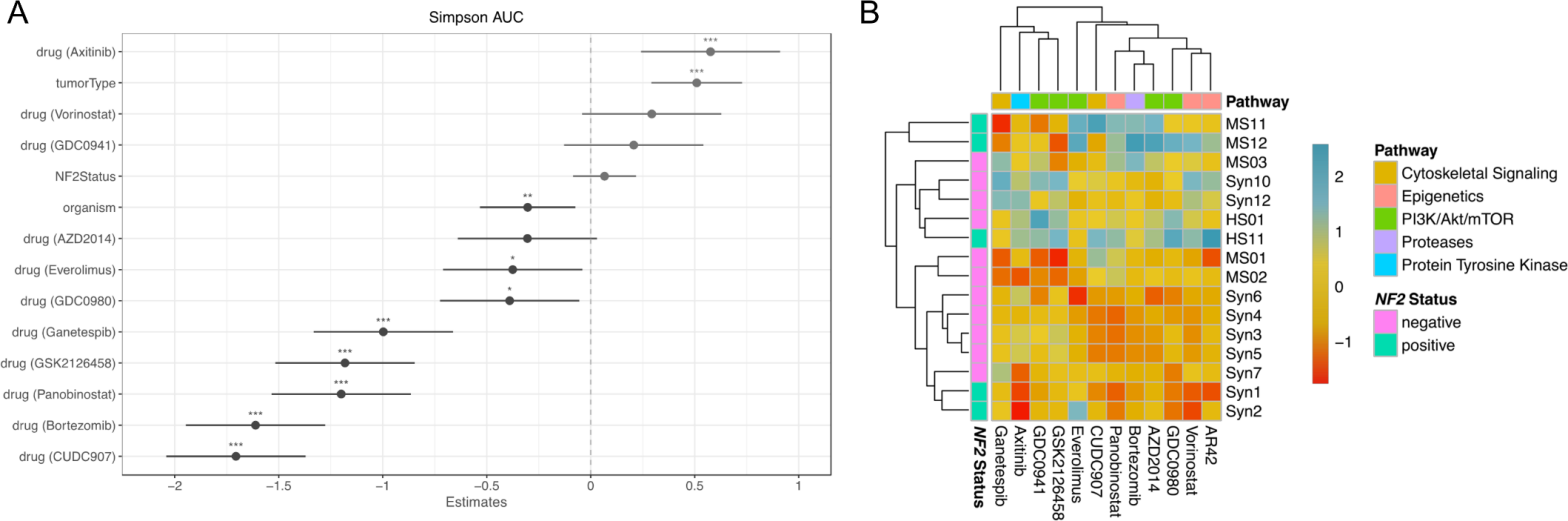
Drug screening outcomes are largely independent of merlin status. **(A)** Estimated beta coefficients of different assay variables to differences in area under the curve (AUC) as determined by multiple linear modeling. Multiple linear modeling indicates that unlike merlin status, tumor type and treatment with a subset of drugs (CUDC-907, Bortezomib, Panobinostat, GSK2126458, Ganetespib, and Axitinib) are significantly associated with a reduction in cell viability as measured by Simpson AUC. **(B)** Hierarchical clustering demonstrates similarity of response (Simpson AUC) of all cell lines to tested drugs. Only drugs common to both cell types were included in the analysis. Based on the response of each cell line to the entire panel of drugs, the cell lines appear to cluster by cell type and merlin status.

We assessed the effects of tumor type, cell line species, and merlin status as covariates for drug response via multiple linear regression (Fig 3; S3 Table), [43]. This method estimates the β coefficient (the slope of the relationship between variables) for each covariate as it pertains to the dependent variable, and calculates the significance of the estimates by performing an *F-*test (testing the null hypothesis the β coefficient is zero). Among these three covariates, tumor type showed the greatest correlation with drug efficacy as measured by area under the curve (AUC) of each drug’s dose response curves (Fig 3A). Under the conditions tested, cell line species showed a moderate correlation with AUC, with mouse lines exhibiting slightly greater drug sensitivity. Hierarchical clustering also showed schwannoma models to be consistently more sensitive than meningioma models (Fig 3B). Conversely, merlin status did not significantly affect drug sensitivity with this panel of drugs (Fig 3A and 3B).

### *In vivo* testing of candidate compounds in a meningioma xenograft model

**Fig 4:**
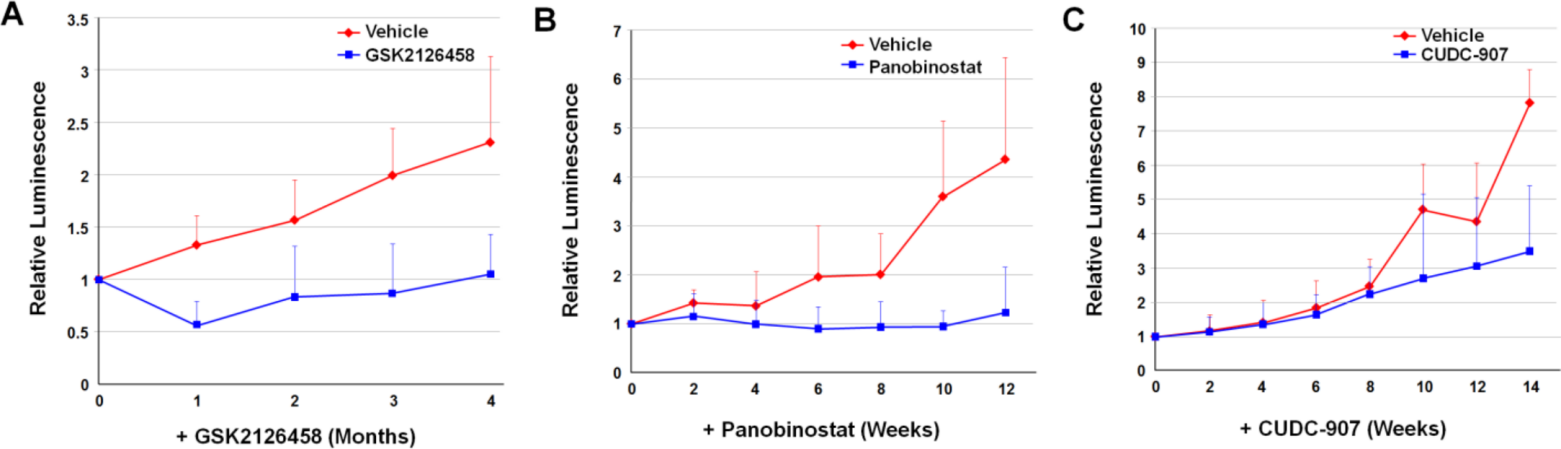
Anti-tumor efficacies of GSK2126458, Panobinostat, and CUDC-907 in the quantifiable, orthotopic, merlin-deficient benign meningioma model. Luciferase-expressing Ben-Men-1-LucB cells were stereotactically injected at the skull base, and mice with established tumors were randomized into three groups for treatment by oral gavage as described in Methods. **(A)** One half of the first group of mice was treated with GSK2126458 and the other half fed the vehicle used for drug formulation (n = 8 each). (**B-C)** Likewise, the second group of mice was treated with **(B)** Panobinostat or the vehicle, and the third group was fed CUDC-907 or **(C)** the formulating vehicle. Tumor growth was monitored by BLI and the relative bioluminescence signals emitted from tumors in mice were quantified and denoted as the percentage of total flux after treatment relative to the total flux prior to treatment designated as one (100%). Shown are the data as mean ± standard deviation.

GSK2126458, Panobinostat, and CUDC-907 were evaluated in the orthotopic merlin-deficient benign meningioma Ben-Men-1-LucB xenograft model [18]. The maximal tolerated doses in this model were determined to be 2 mg/kg/day for GSK2126458 by oral gavage, 20 mg/kg every other day for Panobinostat via intraperitoneal injection, and 25 mg/kg/day for CUDC-907 by oral gavage. Following treatment, tumor growth was monitored by bioluminescence imaging (BLI). Previously work showed that bioluminescence signal in luciferase-expressing tumor xenografts correlates with the tumor size [27]. Mice had a reduction in tumor size within one month of starting GSK2126458. Of note, at six to eight weeks of treatment there was a gradual rebound in tumor-emitted bioluminescence, but even with this, GSK2126458 suppressed tumor growth by an average of ~56% relative to vehicle-treated tumors at four months (Fig 4A). Meningioma xenografts treated with Panobinostat did not grow following treatment, and the magnitude of growth suppression became more significant over time versus vehicle-treated tumors which grew more than 400% (Fig 4B). CUDC-907 at 25 mg/kg/day resulted in a trend of suppression of tumor growth compared with vehicle-treated controls after ten weeks with an average reduction of 55% in tumor size after 14 weeks of treatment (Fig 4C). Growth suppression via in tumor-emitted bioluminescence was corroborated by immunohistochemical analysis (S1 Text).

### *In vivo* testing of candidate compounds in a genetically-engineered mouse schwannoma model

**Fig 5:**
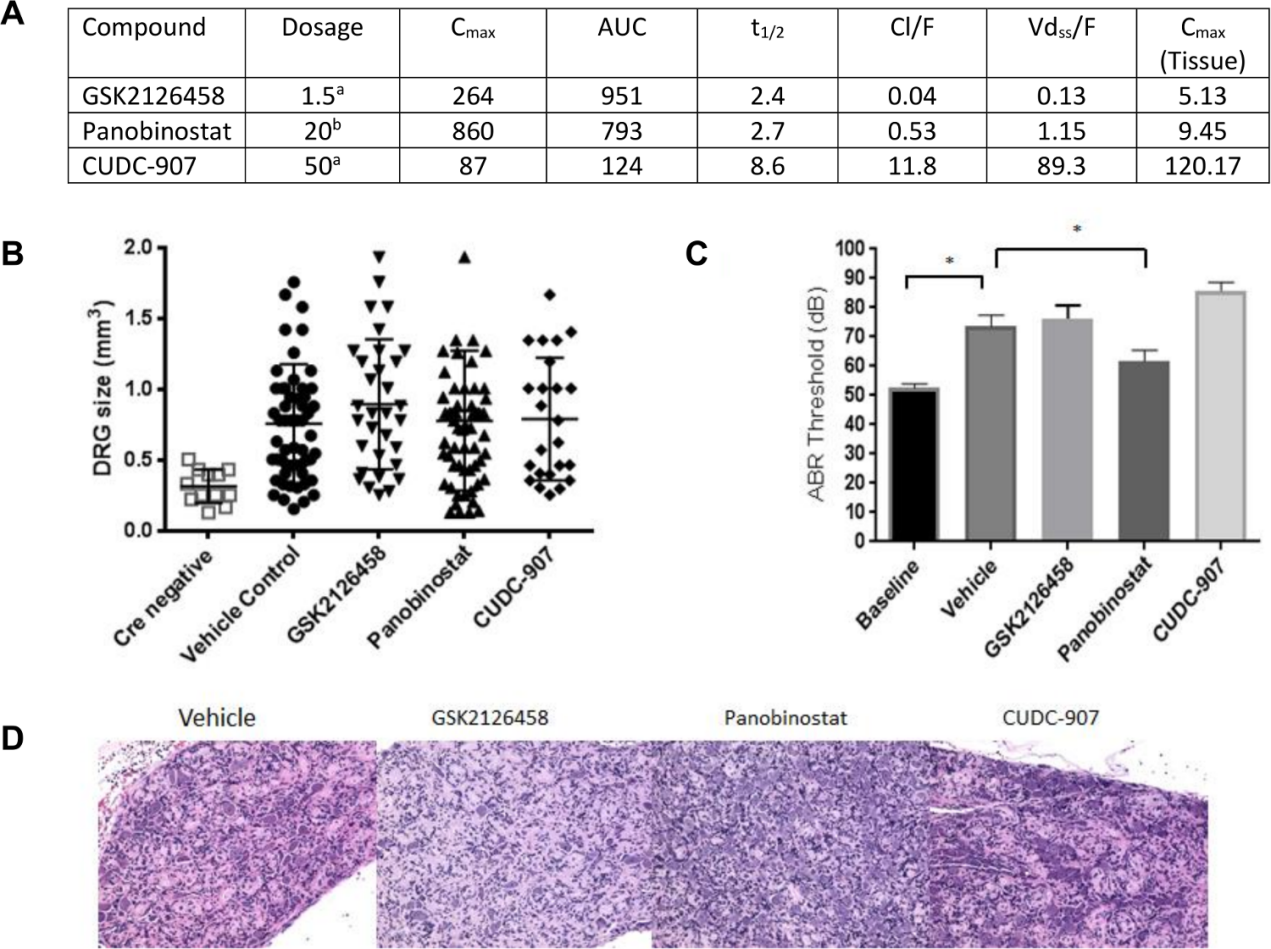
*In vivo* testing in schwannoma. *Nf2* ^*flox/flox*^; *Postn* cre+ mice were treated for 12 weeks at the maximum tolerated dose, beginning at 6 months of age. **(A)** Pharmacokinetics of GSK2126458, Panobinostat and CUDC-907 in *PostnCre; Nf2*^*floxflox*^ mice. Dosage is reported in (a) PO mg/kg/day, (b) IP mg/kg 3x/week. C_max_ is reported in ng/ml. AUC reported as ng/ml/h. t_1/2_ reported in hours. Cl/F reported as L/Hr. Vd_ss_/F reported as L. C_max_ tissue reported as ng/g of tissue. **(B)** Dorsal root ganglion (DRG) size was measured by dial caliper; each data point represents one individual DRG location. Four individual DRG locations were measured per mouse; **(C)** ABR threshold was measured before and after 12 weeks treatment. ABR threshold was significantly increased after 12 weeks with treatment (p>0.0001) by one-way ANOVA. **(D)** Paraffin-embedded slides of DRG from treatment mice were stained with hematoxylin and eosin (H&E).

GSK2126458, Panobinostat, and CUDC-907 were evaluated in a genetically-engineered mouse (GEM) model of NF2, *PostnCre; Nf2*^*floxflox*^, that develops schwannoma of the dorsal root ganglia (DRG) and cranial nerves [19]. Each drug was administered for a treatment period of 12 weeks. Tumor burden and hearing were assessed at the conclusion of treatment. Pharmacokinetic parameters, including tissue concentrations (Fig 5D), were observed to be similar to previously published results [44–46]. GSK2126458 was initially administered by daily oral gavage at 3 mg/kg/day, but subsequently decreased to 1.5 mg/kg/day due to significant GI toxicity. The reduced dose was well tolerated. Panobinostat was given via intraperitoneal injection at a dose of 20 mg/kg 3x/week and was well tolerated. CUDC-907 was poorly tolerated, both at oral doses of 100 mg/kg/day and 50 mg/kg/day pointing to differences with the meningioma model (S2 Text). A dose of 25 mg/kg/day was tolerated. At postmortem examination, dorsal root ganglia at each spinal level were dissected microscopically. Median tumor size was not reduced in response to any of the three experimental drugs (Fig 5B). Further, histologic examination of DRGs from both control and drug treated *PostnCre; Nf2*^*floxflox*^ mice had comparable disrupted architecture consistent with schwannoma irrespective of treatment (Fig 5D). Finally, there was a progressive decline in hearing threshold as measured by ABR regardless of drug used (Fig 5C).

### Molecular characterization of untreated and treated cell lines

A major goal of this work was to investigate the outcomes of traditional drug screening of candidate drugs for tumor toxicity based on phenotype sequentially *in vitro* and *in vivo* as well as to explore the potential for drug discovery driven by molecular and protein characterization via global gene expression and kinome analysis. We hypothesized that the latter might reveal molecular differences that could point to specific therapeutic targets not previously apparent with toxicity specific to merlin-deficient cells. Accordingly, the most reliable and reproducible lines from the meningioma and schwannoma assay systems were selected for transcriptome and kinome analysis. The seven meningioma-relevant and schwannoma-relevant cell lines were treated for 24 hours either with DMSO alone or with the doses of GSK2126458, Panobinostat, CUDC-907 chosen as described in Materials and Methods (S5 Text) and harvested for transcriptome and kinome analyses.

### Transcriptome analysis of meningioma and schwannoma cell systems

**Fig 6:**
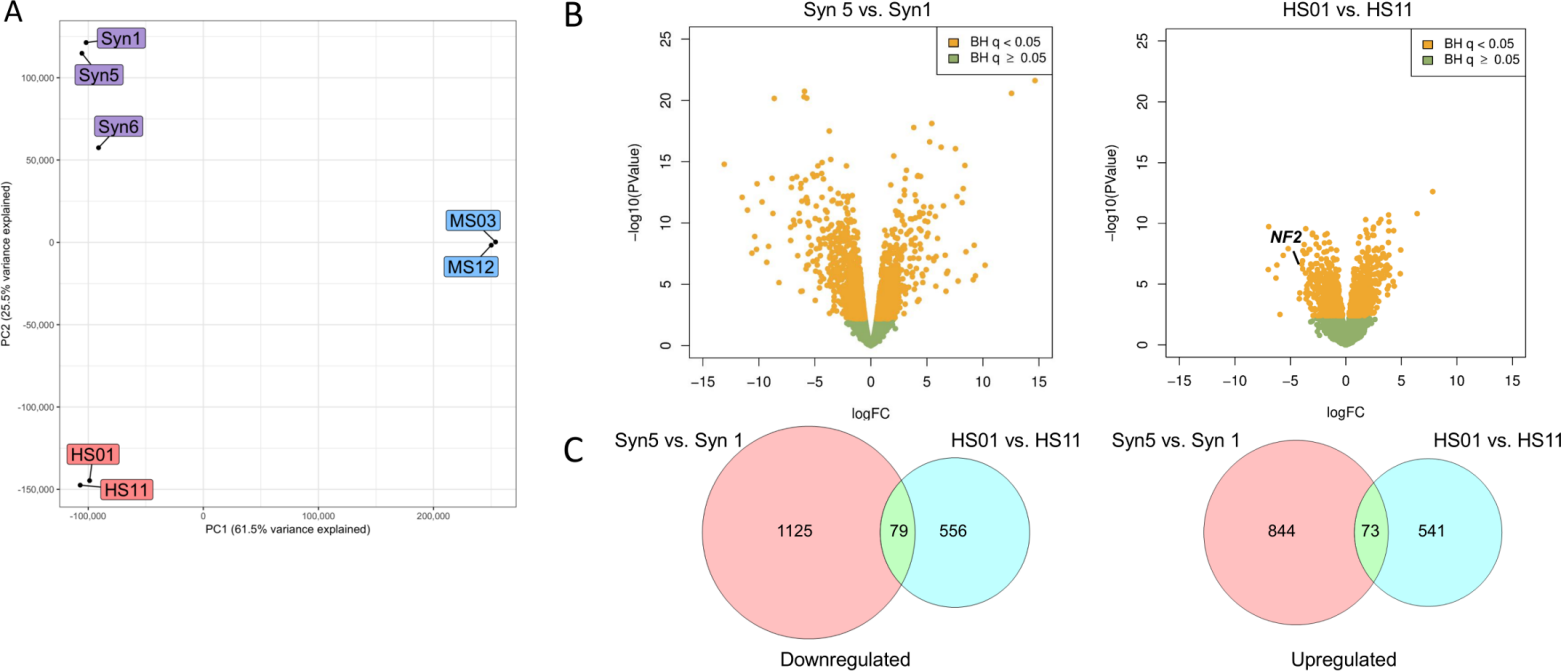
PCA and transcriptomic differences due to merlin deficiency in human AC and SC. **(A)** Principal components analysis (PCA) of averaged, rank-normalized read counts (overlapping genes only) from RNA-seq of wild type and merlin-deficient human SC (red), mouse SC (orange), human AC (blue, Syn5, Syn1) and human MN cells (blue, Syn6). PC1 explains 61.5% of variance, while PC2 explains 25.5% of variance. **(B)** Volcano plots showing the significance and log2 fold-change (logFC) due to merlin deficiency for all gene transcripts reliably detected in the RNA-seq analysis. Yellow dots represent genes altered at [47] adjusted significance of P<0.05. The location of the downregulated *NF2* gene, corresponding with ~7% of normal *NF2* expression, is labelled for HS01 vs HS11. In the Syn5 vs Syn1 AC comparison, fold-comparisons across the entire gene are not meaningful as there is no significant difference in the level of *NF2* transcripts in Syn5, but these produce no active merlin due to absence of exon 8 (S5 Fig), which was removed by CRISPR/Cas9. **(C)** Venn diagrams showing the relatively small degree of overlap between the downregulated (left) and upregulated genes (right) due to merlin deficiency in human AC and SC, respectively.

To assess the effects of merlin deficiency on global gene expression in the seven test cell lines, we performed RNA sequencing (RNAseq). Principal components analysis (PCA) of rank-normalized gene counts indicated that the three models (human AC/MN cells, human SC, and mouse SC) showed substantial transcriptional differences between species and tumor type (Fig 6A); concordant with the response of the cells to the drug panel tested (Fig 3, S3 Table). Direct comparison of the merlin-deficient cells with their merlin-expressing counterparts revealed extensive changes in gene expression (Figs 6B and 6C, S4 and S5 Tables, S3 Text).

Pathway analysis of gene ontology (GO) terms [48] with the Database for Annotation, Visualization and Integrated Discovery (DAVID) [49,50] showed that the human differentially expressed genes point strongly to significant alterations in the extracellular region, plasma membrane and adhesion of both merlin-deficient AC and SC (S6 Table). Some GO terms (such as GO:0005886~plasma membrane, GO:0005887~integral component of plasma membrane, GO:0005615~extracellular space, GO:0005576~extracellular region, and GO:0016021~integral component of membrane) were significantly enriched among both downregulated and upregulated genes in the AC comparison. The same five terms were the most significant among the downregulated genes in human SC, and three of the five were also significant among the upregulated human SC genes. Despite this apparent overlap in the processes disrupted by merlin loss in the two cell types, the actual differentially expressed genes in the two cell systems were largely distinct, with only 240 (21.7%) of the 1,107 differentially expressed genes in the human SC system also being differentially expressed in the human AC system. Off these, only 154 (13.9%) were altered in the same direction (80 down and 74 up) (Fig 6, S7 Table). Similarly, despite the lack of overlap noted above between differentially expressed genes in mouse SC compared to human SC, the mouse differentially expressed genes point to many of the same GO terms as being enriched (S6 Table). The relative lack of correspondence of the actual differentially expressed genes yet enrichment overall of differentially expressed genes for similar GO terms is consistent with loss of merlin function operating in similar processes within the distinct biology of the different target cell types.

A comparison with the druggable genome indicates that many of the differentially expressed genes might be expected to confer differential response or sensitivity of merlin-deficient and wild-type human cells. Of the 1,969 and 1,107 genes differentially expressed due to merlin loss in the human AC and SC comparisons, 343 and 236, respectively, are considered “druggable” based upon the Drug Gene Interaction database (http://dgidb.genome.wustl.edu/) (S8 Table) and many of these are associated with known drugs. For example, as a result of merlin loss, AC overexpress mRNA for the tyrosine kinase EPHA2, which is sensitive to Dasatinib[51], whereas SC overexpress EPHA5 transcripts. Both the AC and SC overexpress *KIT*, encoding the KIT proto-oncogene receptor tyrosine kinase, which is inhibited by Sunitinib [52,53], among many other compounds. Beyond these and several other kinases, there are many other categories of druggable genes whose mRNAs are either over- or under-expressed in merlin-deficient AC and/or SC. Notably in AC, the matrix metallopeptidases, *MMP1*, *MMP2* and *MMP10* and the ion channels, *CACNA1A* and *TRPV2*, and in SC, the G protein-coupled receptors *GPR37* and *NTSR1*, are among the genes most highly upregulated by loss of merlin. Several other categories of potentially “druggable” genes, including transporters, growth factors, proteases, lipases, phosphatases, cell surface proteins, histone modifiers and transcription factors are also represented and provide a variety of potential routes to differential perturbation of wild-type and merlin-deficient human AC and/or SC.

### Transcriptome analysis of drug-treated arachnoidal/meningioma and Schwann cells

Despite these substantial differences between the transcriptomes of merlin-deficient and merlin-wildtype cells, the three drugs that were chosen from our cellular screening for further testing produced no significant differential effect in our cell viability assays, suggesting that they do not target processes that are preferentially important to viability of merlin-deficient cells. However, they did elicit extensive changes in gene expression in the human cell lines (S4 and S5A Tables, S6A Fig), with some hints of differential response that reflect biological differences due to merlin loss (S4 Text; S9 Table).

### Kinome analysis of untreated cells

To understand dynamic changes in the functional kinome, we employed multiplexed Type I kinase inhibitor bead (MIB)/mass spectrometry (MS) kinome profiling. MIBs have preferential affinity for the activated form of kinases, and MIB-binding is dependent on affinity, expression, and activation state of the kinase [54,55]. Gravity-flow captured kinases are eluted and identified by MS. Analyzing isogenic cell lines that differ by a single mutation or comparing samples before and after drug treatment provide a global view of changes in the kinome activation state due to genotype or drug perturbation.

**Fig 7:**
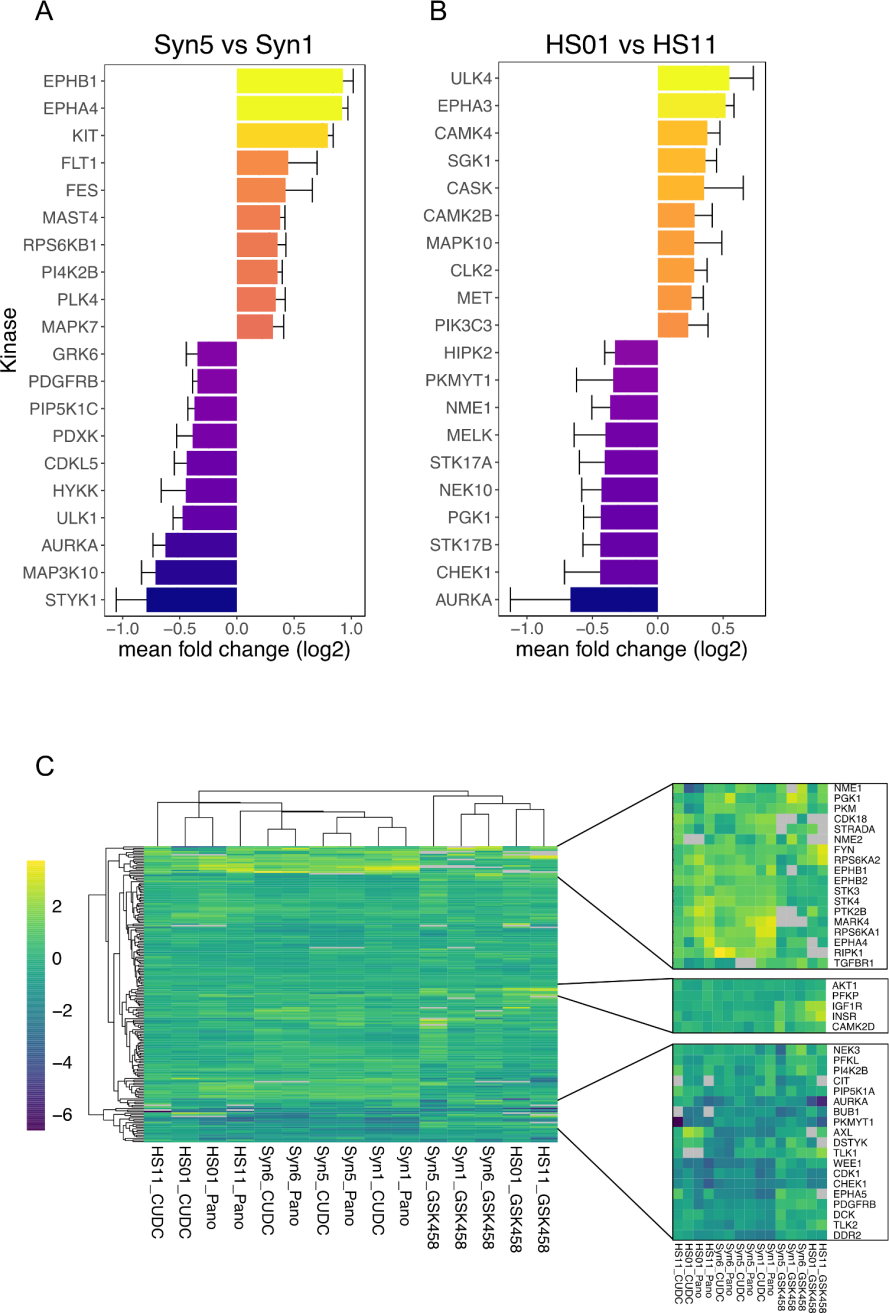
Kinome changes across human isogenic merlin-wildtype and -deficient AC and SC pairs. **(A-B)** Top baseline kinome changes across human AC and SC isogenic pairs. Data presented are the mean log2 fold change from 3 experiments, error bars are standard error. **(C)** Pan-kinome (left) drug induced perturbations relative to DMSO control contain clusters of induced/repressed kinases (right). Each condition is the median log 2 fold change of three replicate experiments relative to vehicle (DMSO) control, with any kinase having >33% non-detection rate removed (grey: kinase not detected in any run). Cell lines treated with HDAC inhibitors (Panobinostat and CUDC-907) cluster most closely.

A comparison of the MIB/MS kinome profiles of merlin wild-type and deficient cells identified several differentially expressed and activated kinases associated with loss of merlin (Fig 7). Among these, merlin-deficient Syn5 AC showed increased MIB binding of receptor tyrosine kinases such as EPH receptor proteins B1 and A4, KIT, and FLT1/VEGFR1, and RPS6KB1/p70S6K as compared to the isogenic merlin-wildtype Syn1 cells. Merlin-dependent differences were also detected in the human SC kinome; the merlin-suppressed HS01 SC line had increased binding of EPH receptor EPHA3, and the Unc51-like kinase ULK4, a serine-threonine kinase with likely function in neuronal growth and migration, and cortex growth [56]. Overall, the baseline data for human meningioma- and schwannoma- relevant isogenic pairs indicate both similarities and differences between these two disease models. Loss of merlin in both cell types results in increased EPH receptor pathway kinases[57], but several non-overlapping kinases associated with merlin-deficient cells were also observed (Fig 7A and 7B). These findings suggest that although some therapeutic approaches may target both meningioma and schwannoma, tumor-type specific targets should also be explored.

In addition to human models, MIB/MS kinome profiles were obtained for a single run of the merlin-deficient (MS03) and merlin-wildtype (MS12) mouse SC lines (S7 Fig). Consistent with the human kinome data, merlin-deficient cells (MS03) displayed increased MIB binding of EPH receptor kinases (EPHB6, EPHA2) as compared to merlin-wildtype mouse SC (MS12).

### Kinome analysis of drug treated cells

We next compared perturbations to the kinome in the meningioma and schwannoma cell systems after treatment with CUDC-907, GSK2126458, Panobinostat, or vehicle. To analyze the effect of all three drugs on the kinome of all three cell line pairs, we also queried a protein interaction database (string-db.org) for potential subnetworks that were selectively induced in the merlin-deficient cell lines.

**Fig 8:**
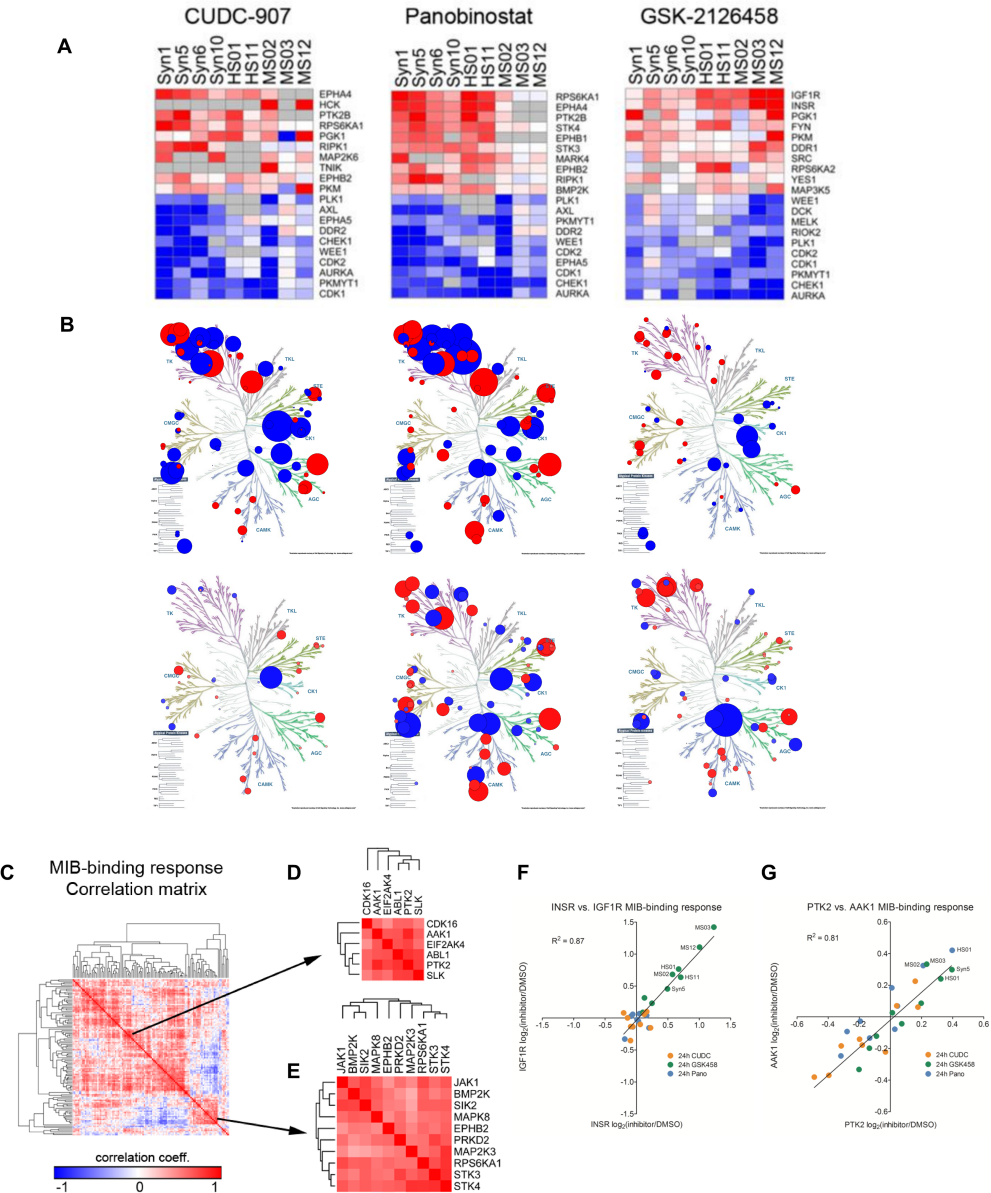
Kinomic response to drug treatment. **(A)** Ranking the top 10 most-induced and most-suppressed kinases for each of the treatments at 24 hours stresses the similarities in drug response across cell lines, and also the differences between human and mouse cell lines. **(B)** Drug induced perturbations in HS-01 and Syn5 shown in a kinome tree plot. **(C)** Kinases that were represented in every cell line were used to generate a MIB-binding response correlation matrix. **(D-E)** Two of the most-prominent clusters include kinases highly-correlated with PTK2 (FAK1) or RPS6KA1 (p90 RSK) and STK3/4. **(F)** The most-highly correlated kinases are INSR and IGF1R (corr = 0.91), primarily being induced by GSK458-treatment at 24 hours. **(G)** The next-most correlated kinase pair across the entire dataset is PTK2 and AAK1 (corr = 0.87). Interestingly, these kinases are preferentially induced in merlin-deficient cell lines.

Initially, we surveyed the drug-specific responses observed by kinome profiling, irrespective of merlin status. Consistent with the growth inhibition observed with the selected compounds, numerous CDKs and mitotic kinases (CDK1, CDK2, AURKA, PKMYT1, PLK1) exhibited dramatic MIB binding loss in all cell lines tested (Fig 8). Dual inhibition of PI3K/mTOR by GSK2126458 led to a strong increase in MIB binding of INSR and IGF1R, which has been previously described in response to mTOR inhibition [58]. Treatment with Panobinostat or CUDC-907 both led to increased MIB binding of PTK2B (FAK2), RPS6KA1 (p90RSK) and the EPH receptors EPHA4 and EPHB2. The shared response between Panobinostat and CUDC-907 could reflect HDAC inhibitor-dependent adaptive changes (Fig 7C) and further suggests that the use of an inhibitor of FAK, RSK, or EPHA4/B2 in combination with Panobinostat and CUDC-907 may be more effective in eradicating merlin-deficient tumors.

Following our assessment of the predominant drug-specific kinome responses, we examined the specific kinome response in the merlin-deficient AC and SC (S8 Fig) versus their merlin-wild-type counterparts. In the meningioma model (Syn5 and Syn1), all three drugs induced merlin-deficient-specific kinase networks that included MAPK proteins, PTK2 (FAK1) and ephrin receptor proteins among other kinases. In the human schwannoma model (HS01 and HS11), PTK2 and PTK2B (FAK1 and FAK2) were induced by all three drugs in a merlin-deficient-specific manner. In addition, both HDAC inhibitors selectively induced mTOR-centered kinase subnetworks in merlin-deficient human SC. We also observed merlin-dependent induced differential kinome perturbation in the mouse schwannoma model (MS03 and MS12). Similar to human schwannoma and meningioma, CUDC-907, GSK2126458 and Panobinostat also selectively induced MAP kinases, ephrin receptor kinases, and PTK2, suggesting that the mouse model recapitulates elements of the human tumor cell response to these drugs.

Overall, HDAC inhibitors generated the largest fold-changes in MIB-binding and at 24 hours clustered separately from GSK458-mediated responses (Fig 7C). The HDAC inhibitors induced a series of tyrosine kinases (TKs) consistently across cell lines, including PTK2, PTK2B, JAK1, FYN, EPHA4, EPHB2, and EPHB1 (Fig 8A).

As several nodes common to the human merlin-deficient models and the three drugs tested were identified, it is possible that cells lacking merlin have a similar kinomic response to treatment with these three drugs. Shown in a kinome tree plot, similarities between CUDC-907-induced and Panobinostat-induced changes were evident (induced changes in Syn5, Fig 8B). Kinases that were represented in every cell line were used to generate a MIB-binding response correlation matrix (Fig 8C–8E). Two of the most-prominent clusters included kinases highly-correlated with PTK2 (FAK1) or RPS6KA1 (p90RSK) and STK3/4. The most-highly correlated kinases were INSR and IGF1R (corr = 0.91), primarily being induced by GSK2126458-treatment at 24 hours (Fig 8F). The next-most correlated kinase pair across the entire dataset was PTK2 and AAK1 (corr = 0.87) (Fig 8G). Interestingly, these kinases were preferentially induced in merlin-deficient cell lines.

To further corroborate the findings from the kinome analysis, Western blot experiments were performed. Merlin-deficient human SC compared to isogenic wild-type human SC have an increased basal level of PTK2B (FAK2). In response to treatment with GSK2126458, CUDC-907 and Panobinostat, both human and mouse merlin-deficient SC cells had increased PTK2B levels when compared to wild-type control cells (S9 Fig). The differential response of PTK2B levels in both merlin negative human and mouse SCs might indicate a potential target for combination therapy. Like the cellular kinome analysis, meningioma xenografts treated with these drugs showed increased levels of pFAK (S4E Fig), suggesting similar responses *in vitro* and *in vivo*.

### Integrated kinome-transcriptome analysis of untreated and drug treated cells

**Fig 9:**
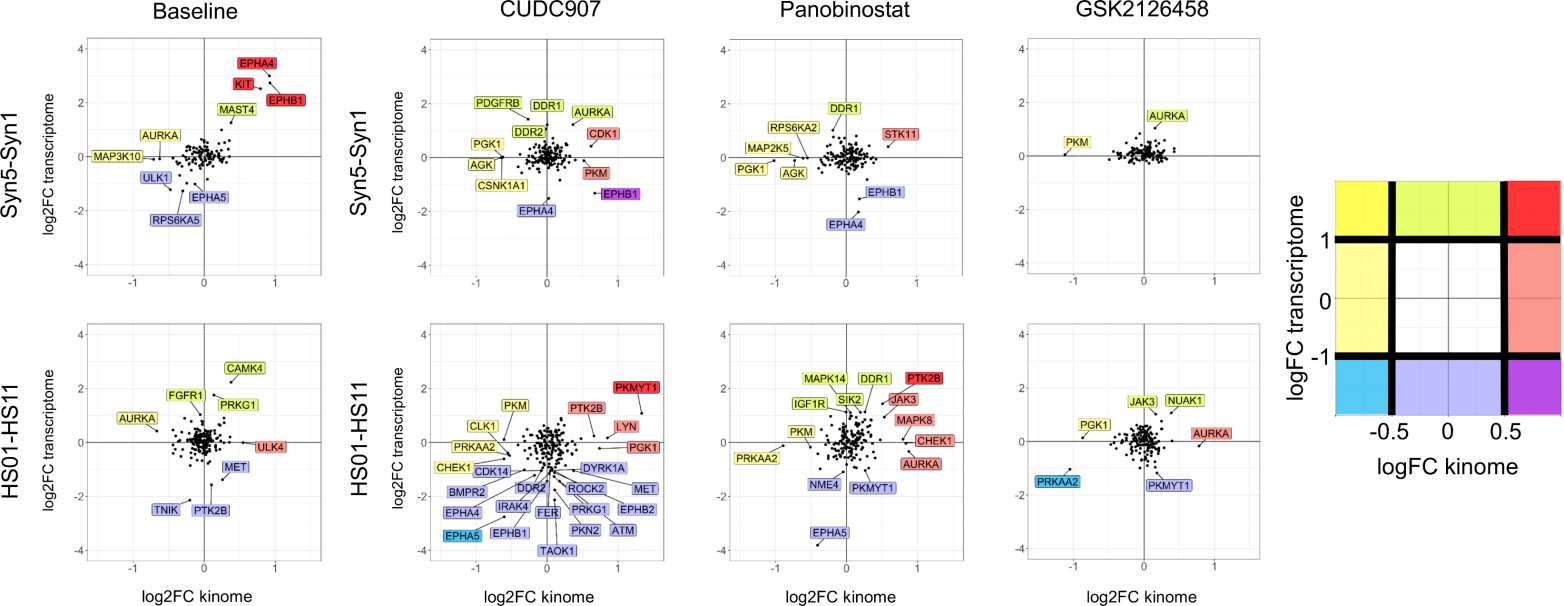
**Integrative analysis of kinome and transcriptome data at baseline (left) and after drug treatment (right) with CUDC-907, Panobinostat, or GSK2126458. In comparisons at baseline or after drug treatment.** Kinases are plotted as black dots based upon the log2 fold difference (logFC) in their activity and transcript level between similarly treated merlin-deficient and merlin-wildtype human isogenic cell pairs, Syn5 vs. Syn1 for AC and HS01 vs. HS11 for SC. Kinases showing a difference of logFC <-0.5 or > 0.5 in kinase activity and/or logFC <-1 or >1 in transcript level are labelled and colored based upon the color code scheme to the right of the graphs (e.g., relatively greater in merlin-deficient cells compared to merlin-wildtype cells than the stated thresholds in both kinase activity and transcriptome data = dark red; relatively greater only in kinase activity = light red, etc.).

To assess the relationship between transcriptome and kinome changes, we performed an integrated analysis of these two datasets at baseline and after CUDC-907, Panobinostat and GSK2126458 treatment of the meningioma- and schwannoma-relevant cell models. In particular, we focused on a comparison between merlin-deficient cells and merlin-wildtype cells for kinases showing a log2 difference of greater than 0.5 or less than -0.5 in the kinome, and/or greater than 1 or less than -1 in the transcriptome (labeled in Fig 9). AC lines without merlin (Syn5 vs Syn1) at baseline showed upregulation of the kinases EPHA4, EPHB1 and KIT in both the kinome and transcriptome. By contrast, in the SC (HS01 vs. HS11), no kinase met the threshold criteria above for having been upregulated in both the kinome and transcriptome (Fig 9, left). No kinases showed downregulation by merlin deficiency in either kinome or transcriptome at these thresholds in either AC or SC, but AURKA showed decreased activity (<-0.5 logFC) due to merlin-deficiency in both cell types without exceeding the threshold difference in the transcriptome. A number of kinases did not show corresponding differences in their activity and transcript levels, such ULK4 in AC which exhibited increased activity without a change in mRNA and PTK2B and MET which were decreased in the transcriptome data (<-1 logFC) but mildly increased in the SC kinome by merlin deficiency.

We also studied the correlation of merlin-dependent changes in the kinome and transcriptome of AC and SC due to drug treatment (Fig 9, right). Unlike the baseline comparison, this analysis was not aimed at whether individual kinase activities or transcript levels increased or decreased *per se* (as presented for merlin-deficient cells in S5 Table, and S6 and S8 Figs). Rather, we assessed relative quantitative differences between the kinome and transcriptome responses of merlin-deficient and merlin-wild-type cells to these drugs. A comparison of fold-changes in these measures in the isogenic pairs reinforced the difference in response of AC and SC to these drugs and the fact that differences in kinase activity often occur in the absence of comparable differences in expression of the kinase-encoding gene. The most striking example of the latter is EPHB1 in CUDC-907 treated AC, where absence of merlin resulted in relatively higher kinase activity (>0.5 LogFC) but relatively lower *EPHB1* gene expression (<-1 LogFC) in Syn5 compared to Syn1. A number of kinases displayed relatively higher activity in the absence of relative differences beyond the threshold for mRNA levels (e.g., AURKA in both Panobinostat and GSK2126458 treated SC). Overall, the integrated analysis suggests that the kinome and transcriptome measures provide somewhat different windows on the response of cells to drug treatment and that both together and separately, they can reveal cell type and genotype (merlin status) differences that can provide additional targets for single or combination drug treatment.

## Discussion

Schwannomas and meningiomas are the most common brain tumors in humans world-wide[3] and despite years of efforts, there are no effective drug therapies for these tumors. In the setting of NF2, the number and distribution of these tumors results in cumulative morbidity and ultimately mortality that cannot be successfully managed with surgery or radiotherapy. As a result, there is a desperate need for new therapeutics for NF2 associated tumors. A partial explanation for the difficulty in identifying effective therapies for NF2 associated schwannomas and meningiomas is the difficulty in creating assay systems given that these are benign, slow growing tumors with relatively bland mutational backgrounds. Moreover, unlike the search for highly cytotoxic compounds aimed at killing aggressive, malignant tumors, NF2 patients require drugs that are well tolerated for long intervals of treatment and inhibit mutant but not normal cells. To address these challenges, this vertically integrated project used cultured Schwann cells (SC) and arachnoidal cells (AC), in some cases genetically manipulated to inactivate or suppress merlin expression, to create a comprehensive cell assay system representing both schwannoma and meningioma. The hope was that a single agent would have activity based on merlin status across tumor types making it clinically feasible for patients to take one agent to address both their *NF2* driven schwannomas and meningiomas. However, despite priming the pipeline with 19 drugs chosen for potential relevance to NF2 biology, we observed relatively little difference overall in the sensitivity of merlin-wildtype cells compared with merlin-deficient cells. Instead, the strongest influences on drug efficacy were cell type (AC vs. SC), followed by species of origin (human vs. mouse).

These are important findings that would not have been possible without assembling a robust, isogenically matched panel of cell lines for the tumors and mutation of interest. The lack of differential drug activity relative to merlin status underscores the inherent difficulty of choosing candidates for testing based upon phenotypic measures alone. While tumor formation is initiated by loss of merlin, many of the consequent characteristics and altered pathways observed in tumors and tumor cells may not be essential for their propagation and therefore may not be useful targets for genotype-specific therapeutic development. An assessment of such features is particularly difficult in primary tumor specimens where a truly comparable normal tissue sample is usually lacking. By establishing a cell-based system with genetically-matched merlin-expressing and merlin-deficient cells representing the cellular targets of the two major tumor types in NF2 patients, we provide the capacity to go beyond the phenotypic candidate approach to strategies informed by genotype-dependent systems analyses or to larger, mechanism-agnostic drug screens.

The cell type and species differences seen in the drug screen were reinforced with the transcriptome and kinome analyses of isogenic pairs of merlin-deficient and merlin-wildtype cells. In the transcriptome studies, the absence of merlin resulted in global differences in gene expression in SC and AC versus their wild-type counterparts. In both human and mouse SC pairs and in the human AC pair, the gene expression changes indicated that loss of merlin function disrupts processes related to the plasma membrane and extracellular region/space. However, the actual genes dysregulated were largely non-overlapping, suggesting that the loss of functional merlin affects shared processes in different ways, in different cell types. This is an important, yet subtle, discovery that will inform drug development strategy for *NF2* driven tumors.

Principal components analysis of the global gene expression data emphasized that cell type and species differences outweigh the effects of merlin deficiency. Similarly, kinome analysis revealed the activation of different kinases in human AC compared to human SC. Taken together, these data suggest that while schwannomas and meningiomas are both initiated by merlin loss, distinctly different responses to that loss likely dictate tumor propagation. Clinically, this means that a single drug is unlikely to be optimally effective in both cell types. Further, when selecting a drug for one tumor type, caution will be required about potential transcriptome and kinome effects on the other tumor type.

In the absence of strong selective toxicity to merlin deficient cells for the 19 drugs tested, we chose three, CUDC-907 (dual PI3K/HDAC inhibitor), Panobinostat (HDAC inhibitor), and GSK2126458 (dual PI3K/mTOR inhibitor) for *in vivo* testing based on traditional markers of activity *in vitro* across all cell lines. We saw activity, notably for GSK2126458 and Panobinostat, in the meningioma *in vivo* model, but none of the three agents was active *in vivo* in the GEM schwannoma model. The difference *in vitro* versus *in vivo* activity for schwannoma may be due to contributions from the microenvironment that are not addressed in either the *in vitro* schwannoma systems or in the allograft meningioma system [59]. It also highlights that the cell-type specific 2D culture system cannot completely reflect either the vulnerabilities or resistances of the corresponding tumor *in vivo*. The lack of strong selective toxicity against merlin-deficient cells with the initial 19 drug candidates screened likely indicates that they all target processes whose role in determining viability *in vitro* is not dependent on merlin, although it cannot be excluded that a role for merlin activity in determining viability in a system more faithful to the 3D *in vivo* environment might reveal selectivity for one or more of these compounds.

Developing advanced assays and screening a much larger panel of chemical compounds are two potential approaches to achieving selective toxicity for the merlin-deficient tumor cells while sparing their wild-type counterparts. Indeed, we would have altered the drug panel screened if had baseline transcriptome and kinome data before starting the drug screen. For example, the emergence of increased activity of ephrin receptor kinases in merlin-deficient cells would have prompted the addition of Dasatinib to the screen and increased CAMK4 in human merlin-deficient SC would have suggest the drug KN-93 [53,60]. In fact, based on the observed changes in kinome in *NF2*-null human AC and MN cell lines in this study a therapeutic trial was undertaken *in vitro* and *in vivo* that indeed showed synergy between dasatinib and a dual mTORC inhibitor that was predicted by kinome results[61]. There are many other options available both among kinases and other known druggable targets. Hence, using the cell lines characterized in this work, efforts are ongoing for more drug screening with more comprehensive drug libraries.

Perhaps the most important outcome of this work was the validation of diverse cell lines with isogenic pairs allowing the generation of transcriptome and kinome data at baseline and in response to drug. This is a rich data source to mine to identify combination therapies that influence transcriptome or kinome; potentially engaging merlin deficiency in determining viability. Combination therapy guided by such molecular data may reveal circumstances in which response to a single agent in merlin-deficient cells creates a vulnerability to be exploited by a second agent, achieving selective toxicity. Future work may use the differences between merlin-wildtype and merlin-deficient cells in their molecular response to CUDC-907, Panobinostat, and GSK2126458, to identify potential agents that will be additive or synergistic. For example, the AMP-activated protein kinase NUAK1 (inhibitor: WZ4003) [62] was selectively upregulated in merlin-deficient schwannoma cells by GSK2126458. PTK2B (inhibitor: defactinib) [63] was upregulated in merlin-deficient schwannoma cells by Panobinostat and AURKA (inhibitor: alisertib) [64] was upregulated in merlin-deficient meningioma upon treatment with GSK2126458 and CUDC-907. Expanding the list of those datasets available to isogenic pairs treated with drugs suggested by the baseline transcriptome and kinome data may provide yet more attractive combinations based upon the emerging biology of merlin-deficiency in these cell types.

The ongoing efforts of the Synodos for NF2 Consortium have already filled a critical gap of understanding of how the *NF2* mutation and the absence of merlin influence tumor cell behaviour in both meningiomas and schwannomas and succeeded in: (1) creating a new pipeline of well-validated cell lines for NF2-associated drug discovery with fully characterized isogenic pairs of meningioma and schwannoma cell systems; (2) gaining conceptual insights into disease mechanisms via transcriptome and kinome interrogation that indicate that different drugs are likely to be needed to treat meningioma and schwannoma; (3) validating pathways and targets that are altered based on tumor type (more so than mutational status) and in response to targeted pathway inhibition in the setting of *NF2;* and 4) building an openly accessible (www.synapse.org/SynodosNF2) database for community sharing and mining of drug treatment, transcriptome and kinome data from isogenic schwannoma and meningioma cell systems. These data already present several targets to consider for single agent and combination therapy in the short term and provide a deep and rich open access data resource for the broader research community seeking treatments for NF2 associated meningioma and schwannoma for the long term.

## Materials and methods

Detailed materials and methods can be found in S5 Text.

## Supporting information

S1 Table Response measurements in AC and MN lines of compounds not meeting efficacy metrics. (XLSX)

S2 Table Response measurements in human and mouse SC/schwannoma lines of compounds not meeting efficacy metrics. (XLSX)

S3 Table Drug screening outcomes are largely independent of merlin status. Multiple linear modeling was used to test the association of each assay variable with differences in area under the curve (Simpson AUC). This table shows the effect of each variable on the AUC as determined by the model (the beta coefficient, B), the 95% confidence interval (CI) for the beta coefficient, and calculated significance (p) for the beta coefficient. Only drugs common to both cell types were included in the analysis. The fraction of observations that are explained by the model (R^2^) and the total number of data points in the model (Observations) are also shown.
(XLSX)

S4 Table Number of differentially expressed genes in merlin-wildtype and merlin–deficient cell lines at baseline and in response to drug treatments. (XLSX)

S5 Table Transcriptomic differences in isogenic pairs of untreated and drug-treated merlin -deficient and merlin-wildtype human arachnoidal cells and Schwann cells (A) and mouse Schwann cells (B)
(XLSX)

S6 Table Gene Ontology (GO) Terms significantly enriched among differentially expressed genes due to merlin deficiency. (XLSX)

S7 Table Genes differentially expressed due to merlin deficiency in both human arachnoidal cells and Schwann cells. (XLSX)

S8 Table Representation in the druggable genome of human genes differentially expressed due to merlin deficiency in human arachnoidal cells and Schwann cells. (XLSX)

S9 Table Differentially expressed genes due to drug treatment of isogenic human merlin-wildtype and merlin-deficient arachnoidal cell and Schwann cell pairs that are discordant for direction of response. (XLSX)

S1 Fig Characterization of screening cell lines. **(A)** Immunoblotting of isogenic immortalized AC-CRISPR clones (iACs) using the N-terminal anti-MERM antibody N21 (raised to a common epitope shared between merlin and other ERM protein family members) shows loss of merlin in Syn3-5 compared to merlin-wildtype Syn2, with intact expression of other ERM family members. **(B*)*** Immunoblotting of representative panels of iACs (AC-CRISPR clones Syn1-5), immortalized MN (iMN, Syn6), and primary MN cell lines (Syn7, Syn10, Syn12) show merlin-deficient (-) compared to merlin-wildtype (+) Syn1 and Syn2 lines. Loading controls included housekeeping proteins ribosomal S6 subunit (left and center panel) and GAPDH (right panel). **(C)** Representative merlin Western blots of whole cell extracts from primary mouse Schwann cells MS11 (WT) and merlin-deficient (MD; *Nf2ex2-/-)* MS01, MS02 lines, isogenic MS12 (WT) and MS03 (MD), and isogenic HS11 (WT) and HS01 (MD). β-actin was immunoblotted as a loading control. **(D)** Confocal images of mouse Schwann /schwannoma cell lines MS11, MS01, MS02, MS12 and MS03 showing the SC marker S-100 (green). Human Schwann cell lines HS11 and HS01 displaying S-100 (green) and human nuclear antigen (HNA, red). DAPI stained nuclear DNA (blue), and F-actin (phalloidin-Alexa633; white) is also shown. Scale bar: 50 μm.
(TIF)

S2 Fig Treatment response of human merlin-wildtype and merlin-deficient cells with compounds failing to meet efficacy metrics. **(A)** Human arachnoidal and meningioma cells. CellTiter-Glo was assessed at 72 hours of drug treatment **(B)** Human Schwann cells. CellTiter-Fluor was assessed at 48 hours of drug treatment with increasing concentration at half-log concentrations, ranging from 0.001 μM to 10 μM.
(TIF)

S3 Fig Treatment response of mouse merlin-wildtype and merlin-deficient cells with compounds failing to meet efficacy metrics. CellTiter-Fluor was assessed at 48 hours of drug treatment with increasing concentration at half-log concentrations, ranging from 0.001 μM to 10 μM.
(TIF)

S4 Fig Immunohistochemistry confirmation of target engagement in Ben-Men1 (Syn6) tumors. **(A)** Acetylated histone lysine was evaluated in Syn6 tumors as a readout of HDAC inhibition. **(B)** pAKT(Thr308 and Ser473) and pS6(S235/236) reduction demonstrate AKT pathway inhibition in Syn6 tumors after treatment with all three drugs. **(C)** pS6(S235/236) and **(D)** Ki67 was reduced in Syn6 tumors after treatment with GSK2126458, Panobinostat, and CUDC-907, while **(E)** pFAK (Tyr397) was increased.
(TIF)

S5 Fig Integrated genomics viewer comparison of *NF2* transcripts from RNAseq in Syn5 and MS03. **(A)** Plotting of transcript reads against the exon structure of NF2 demonstrates the complete skipping of the CRISPR/Cas9-targeted exon 8 and presence of a novel antisense RNA in Syn5 compared with Syn1. **(B)** *Nf2* transcripts show complete skipping of exon 2, a floxed exon removed by Cre recombinase, in MS03 compared with MS12.
(TIF)

S6 Fig Transcriptome response of merlin-deficient human cells to drug treatments. **(A)** Volcano plots showing the significance and log2 fold-change (logFC) for all gene transcripts reliably detected in the RNA-seq analysis after treatment of Syn5 or HS01 with the noted drug, in comparison with exposure to the DMSO vehicle. Yellow dots represent genes altered at BH adjusted significance P<0.05.
**(B)** Venn diagrams showing the overlap between the genes downregulated (left) and upregulated (right) due to the above drug treatments.
(TIF)

S7 Fig LFQ kinome measurements of mouse schwannoma cell line MS03 versus MS12 (single run). (TIF)

S8 Fig Kinome changes in human merlin-deficient arachnoidal cells and Schwann cells. Top kinome changes in merlin deficient human AC (Syn5) and SC (HS01) treated with CUDC-907, Panobinostat or GSK2126458. Data presented are the median log2 fold change from 3 experiments, error bars are standard error.
(TIF)

S9 Fig PYK2 differential response to drug treatment. Both merlin-deficient mouse and human Schwann cells (SCs) increase PYK2 levels in response to DMSO, CUDC-907, Panobinostat and GSK2126458. Immunoblot analysis of human (HS11 and HS01) **(A)** and mouse (MS12 and MS03) **(B)** merlin-deficient SCs compared to wild-type control. Membranes were treated with merlin and PYK2 antibodies as indicated. 25 μg of lysates were used and equal loading was controlled by Ponceau S staining. These immunoblots are representative of three independent experiments.
(TIF)

S1 Text *In vivo* testing of candidate compounds in a meningioma xenograft model–immunohistochemical analysis. (DOCX)

S2 Text Translation of *in vitro* to *in vivo* efficacy studies in meningioma and schwannoma models. (DOCX)

S3 Text Transcriptome analysis of meningioma and schwannoma cell systems–differential gene expression. (DOCX)

S4 Text Transcriptome analysis of drug-treated arachnoidal/meningioma and Schwann cells. (DOCX)

S5 Text Materials and methods. (DOCX)
