# Supplementary figures and images for: Traditional and systems biology based drug discovery for the rare tumor syndrome neurofibromatosis type 2
Source: PLoS One. 2018 Jun 13;13(6):e0197350. doi: 10.1371/journal.pone.0197350 (PMC5999111; doi:10.1371/journal.pone.0197350)

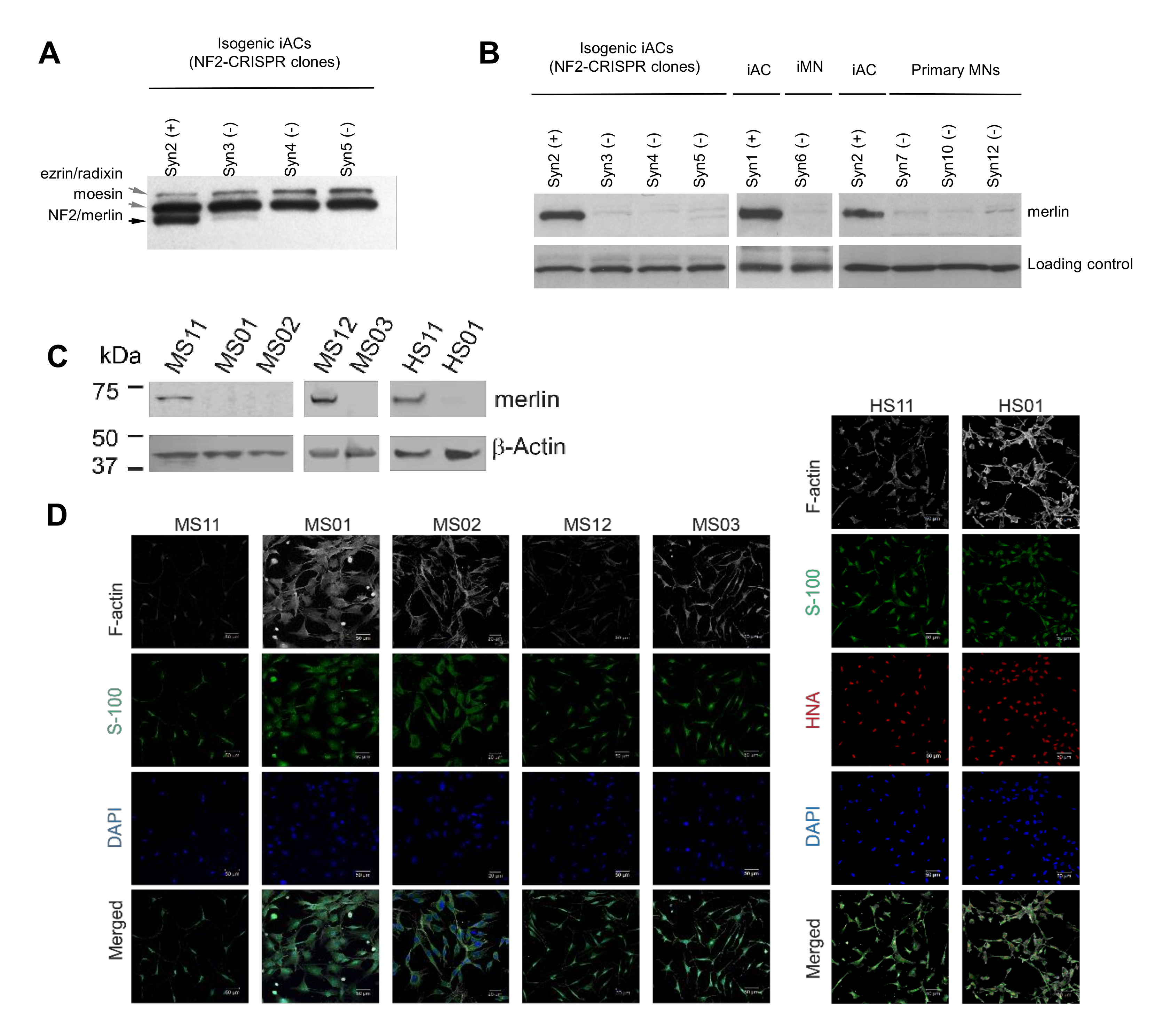

Supplement: S1 Fig — (A) Immunoblotting of isogenic immortalized AC-CRISPR clones (iACs) using the N-terminal anti-MERM antibody N21 (raised to a common epitope shared between merlin and other ERM protein family members) shows loss of merlin in Syn3-5 compared to merlin-wildtype Syn2, with intact expression of other ERM family members. (B) Immunoblotting of representative panels of iACs (AC-CRISPR clones Syn1-5), immortalized MN (iMN, Syn6), and primary MN cell lines (Syn7, Syn10, Syn12) show merlin-deficient (-) compared to merlin-wildtype (+) Syn1 and Syn2 lines. Loading controls included housekeeping proteins ribosomal S6 subunit (left and center panel) and GAPDH (right panel). (C) Representative merlin Western blots of whole cell extracts from primary mouse Schwann cells MS11 (WT) and merlin-deficient (MD; Nf2ex2-/-) MS01, MS02 lines, isogenic MS12 (WT) and MS03 (MD), and isogenic HS11 (WT) and HS01 (MD). β-actin was immunoblotted as a loading control. (D) Confocal images of mouse Schwann /schwannoma cell lines MS11, MS01, MS02, MS12 and MS03 showing the SC marker S-100 (green). Human Schwann cell lines HS11 and HS01 displaying S-100 (green) and human nuclear antigen (HNA, red). DAPI stained nuclear DNA (blue), and F-actin (phalloidin-Alexa633; white) is also shown. Scale bar: 50 μm. (TIF) [file pone.0197350.s010.tif]

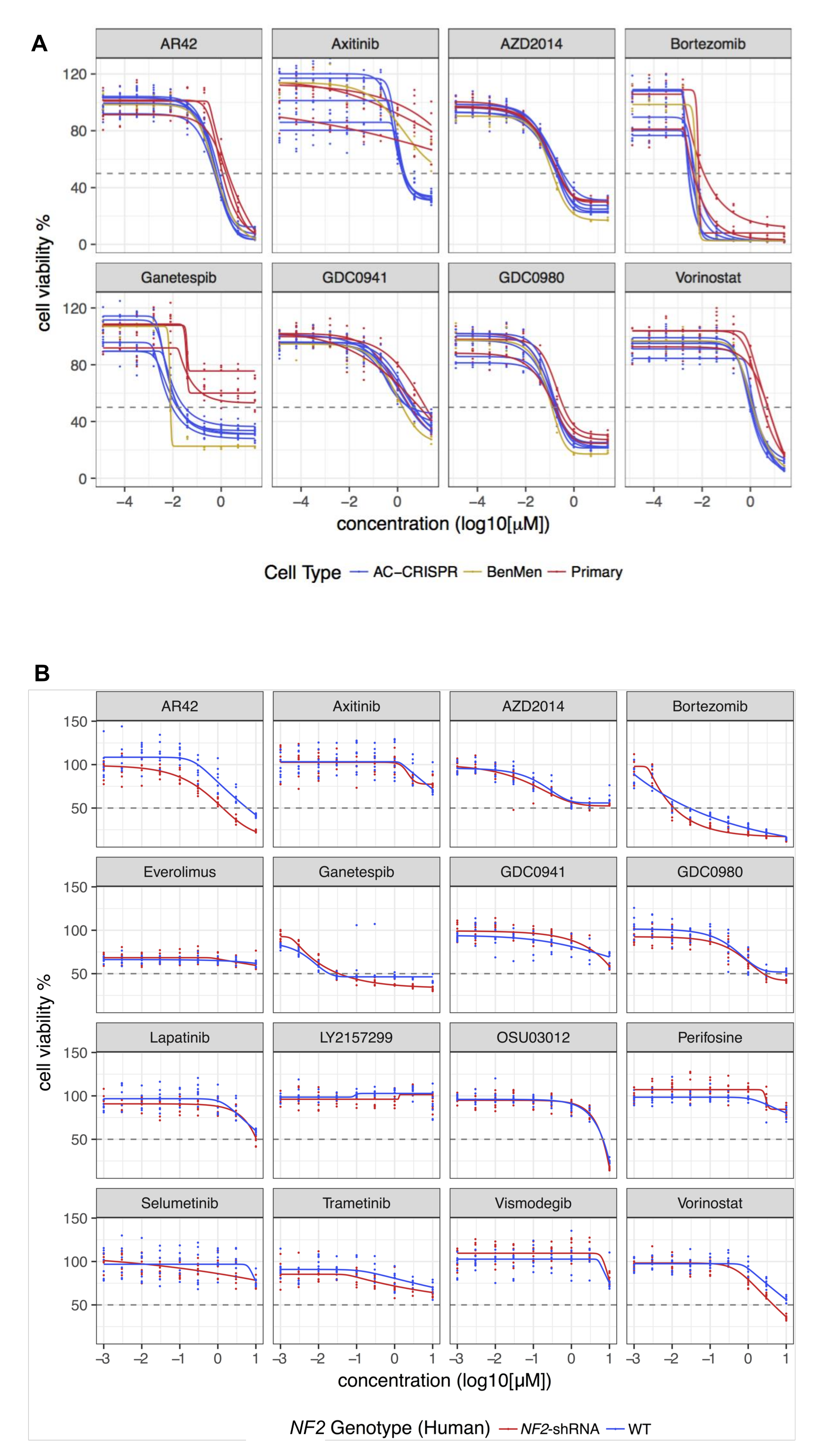

Supplement: S2 Fig — (A) Human arachnoidal and meningioma cells. CellTiter-Glo was assessed at 72 hours of drug treatment (B) Human Schwann cells. CellTiter-Fluor was assessed at 48 hours of drug treatment with increasing concentration at half-log concentrations, ranging from 0.001 μM to 10 μM. (TIF) [file pone.0197350.s011.tif]

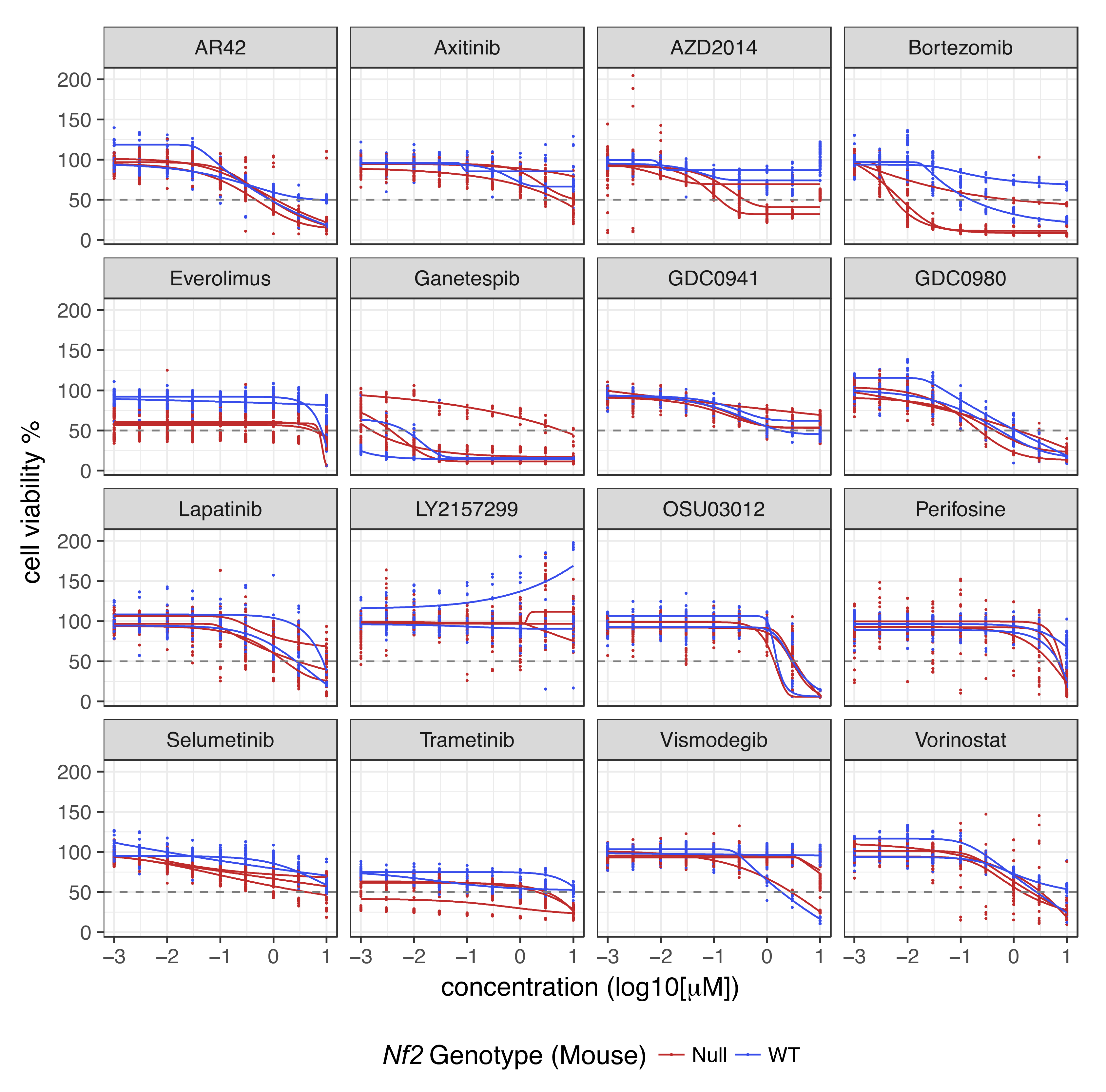

Supplement: S3 Fig — CellTiter-Fluor was assessed at 48 hours of drug treatment with increasing concentration at half-log concentrations, ranging from 0.001 μM to 10 μM. (TIF) [file pone.0197350.s012.tif]

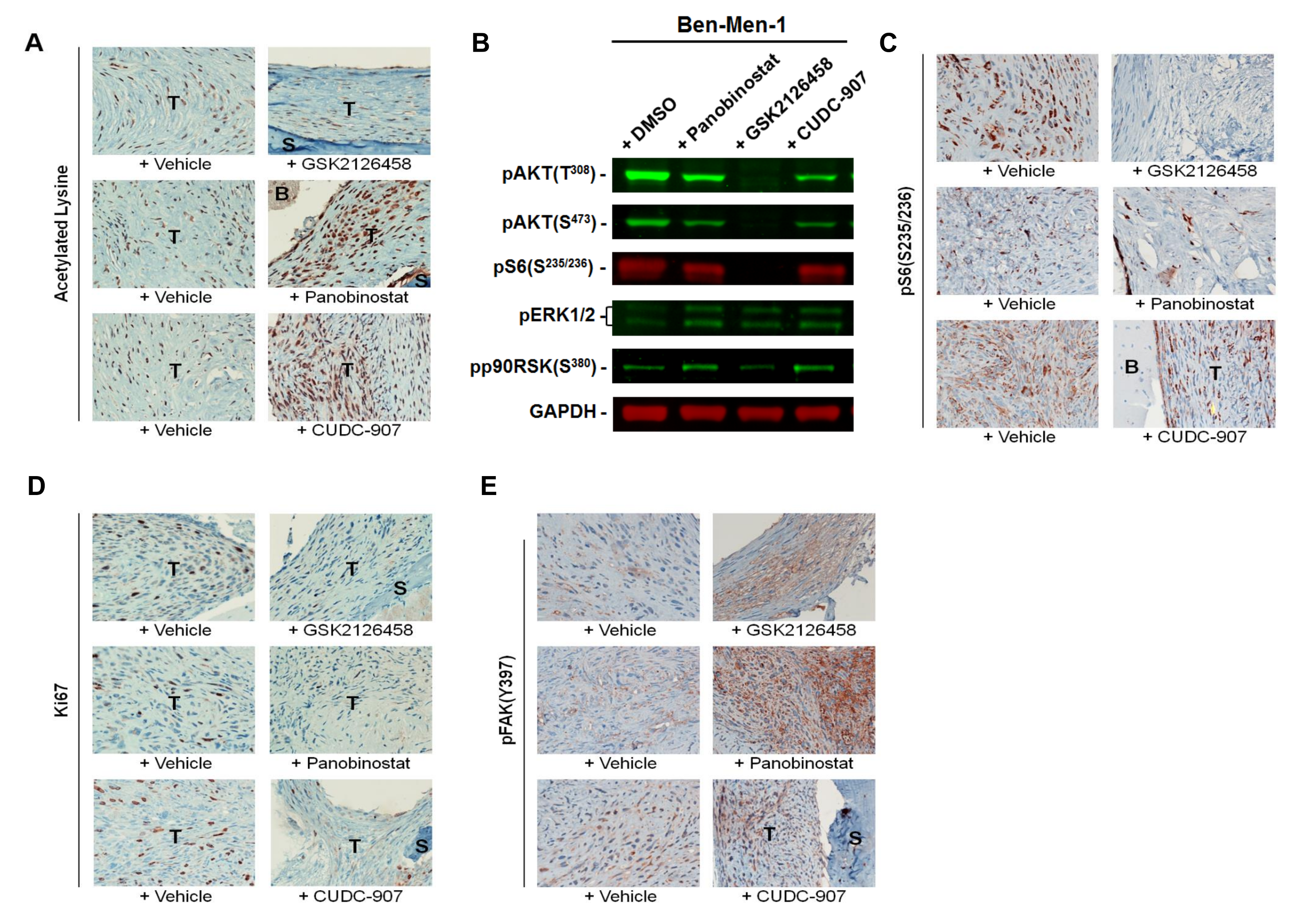

Supplement: S4 Fig — (A) Acetylated histone lysine was evaluated in Syn6 tumors as a readout of HDAC inhibition. (B) pAKT(Thr308 and Ser473) and pS6(S235/236) reduction demonstrate AKT pathway inhibition in Syn6 tumors after treatment with all three drugs. (C) pS6(S235/236) and (D) Ki67 was reduced in Syn6 tumors after treatment with GSK2126458, Panobinostat, and CUDC-907, while (E) pFAK (Tyr397) was increased. (TIF) [file pone.0197350.s013.tif]

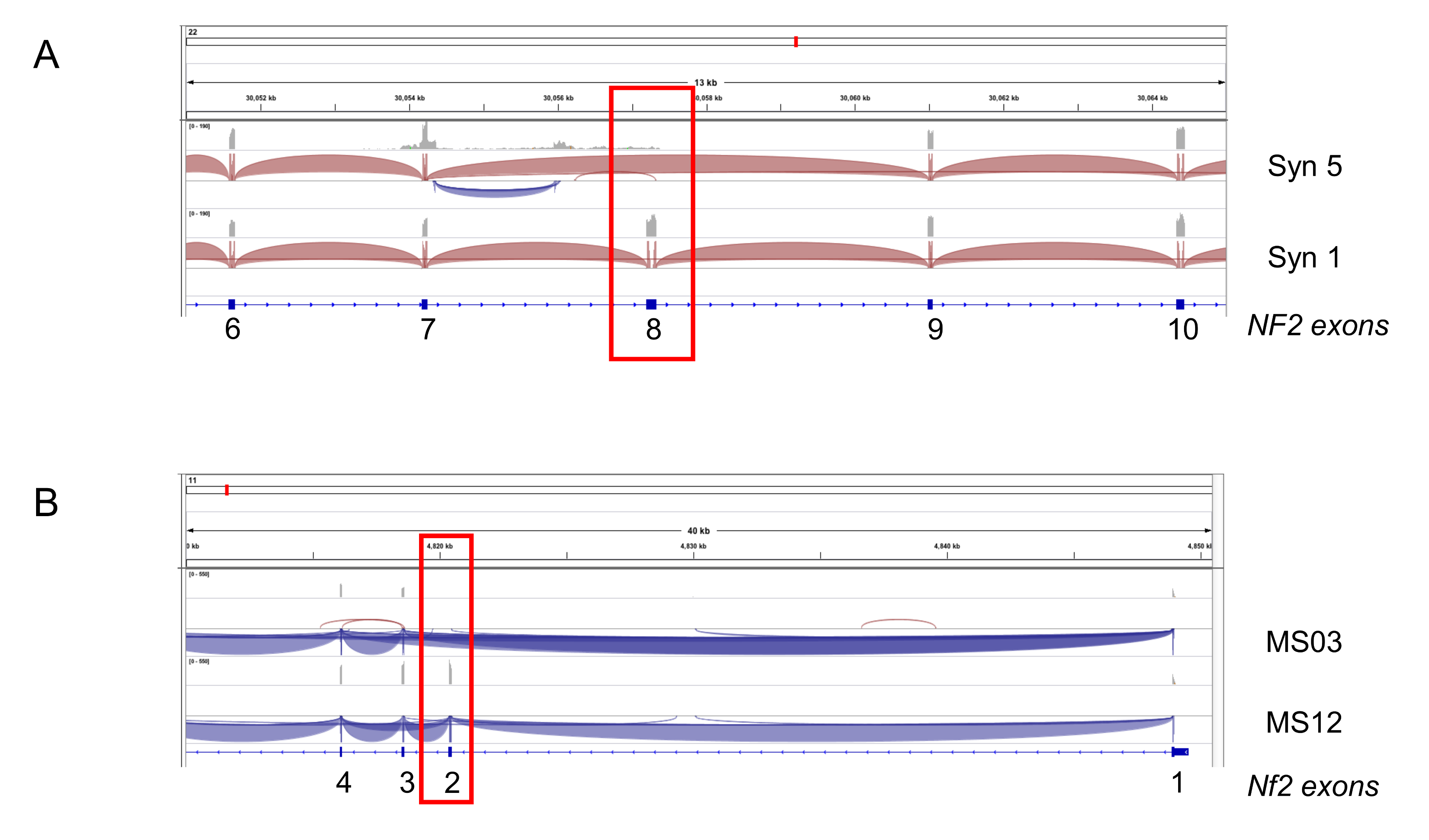

Supplement: S5 Fig — (A) Plotting of transcript reads against the exon structure of NF2 demonstrates the complete skipping of the CRISPR/Cas9-targeted exon 8 and presence of a novel antisense RNA in Syn5 compared with Syn1. (B) Nf2 transcripts show complete skipping of exon 2, a floxed exon removed by Cre recombinase, in MS03 compared with MS12. (TIF) [file pone.0197350.s014.tif]

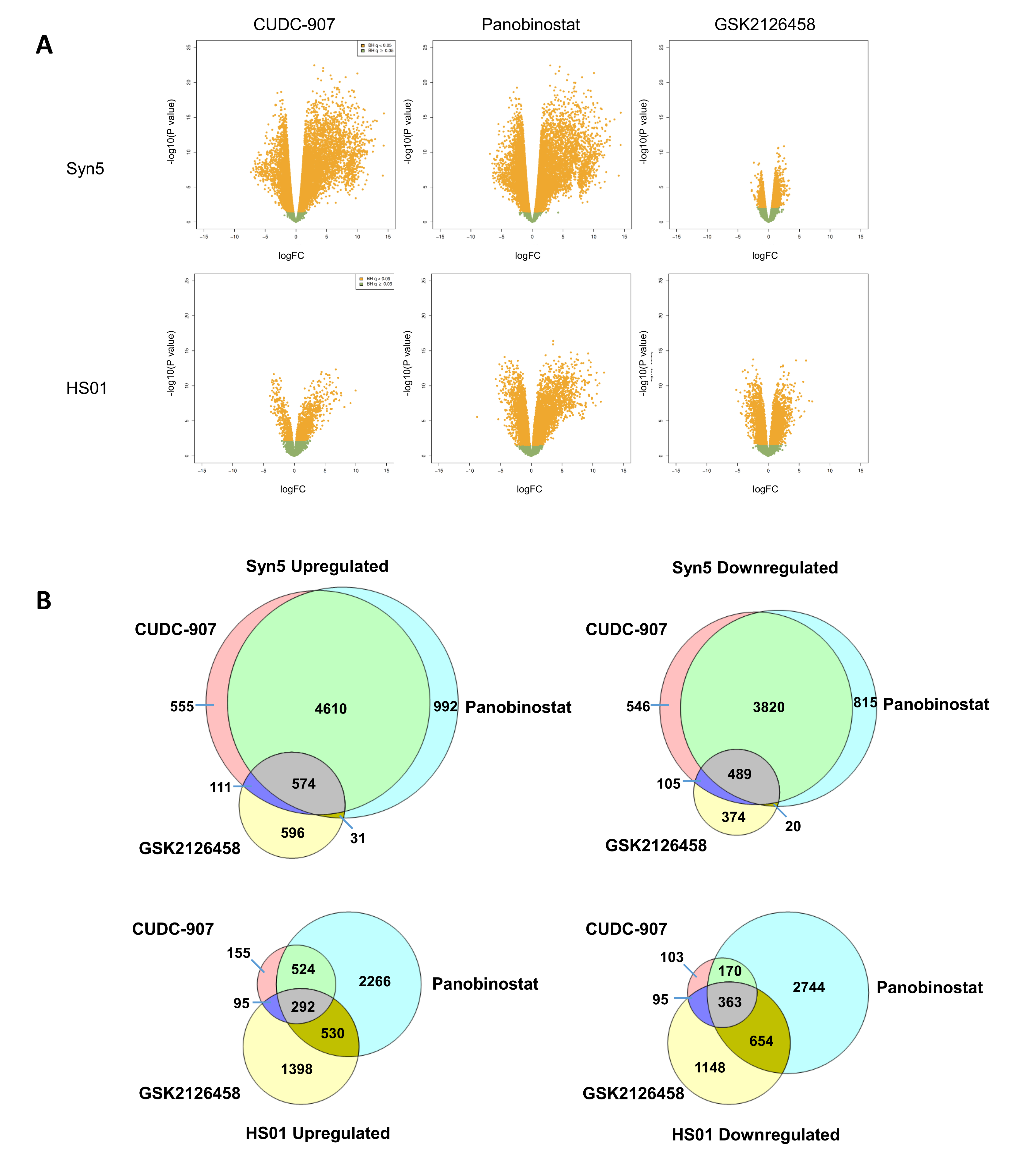

Supplement: S6 Fig — (A) Volcano plots showing the significance and log2 fold-change (logFC) for all gene transcripts reliably detected in the RNA-seq analysis after treatment of Syn5 or HS01 with the noted drug, in comparison with exposure to the DMSO vehicle. Yellow dots represent genes altered at BH adjusted significance P<0.05. (B) Venn diagrams showing the overlap between the genes downregulated (left) and upregulated (right) due to the above drug treatments. (TIF) [file pone.0197350.s015.tif]

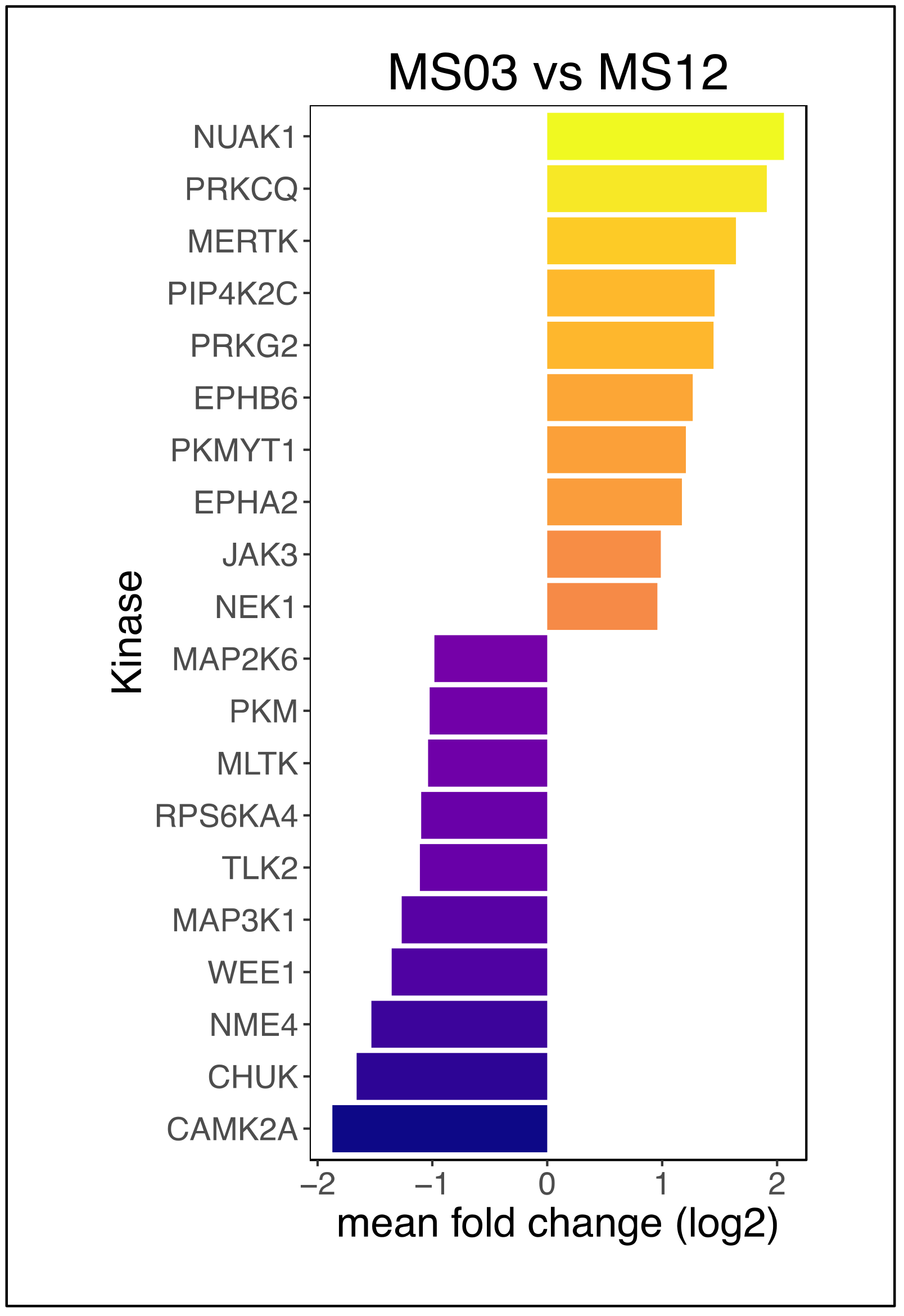

Supplement: S7 Fig — (TIF) [file pone.0197350.s016.tif]

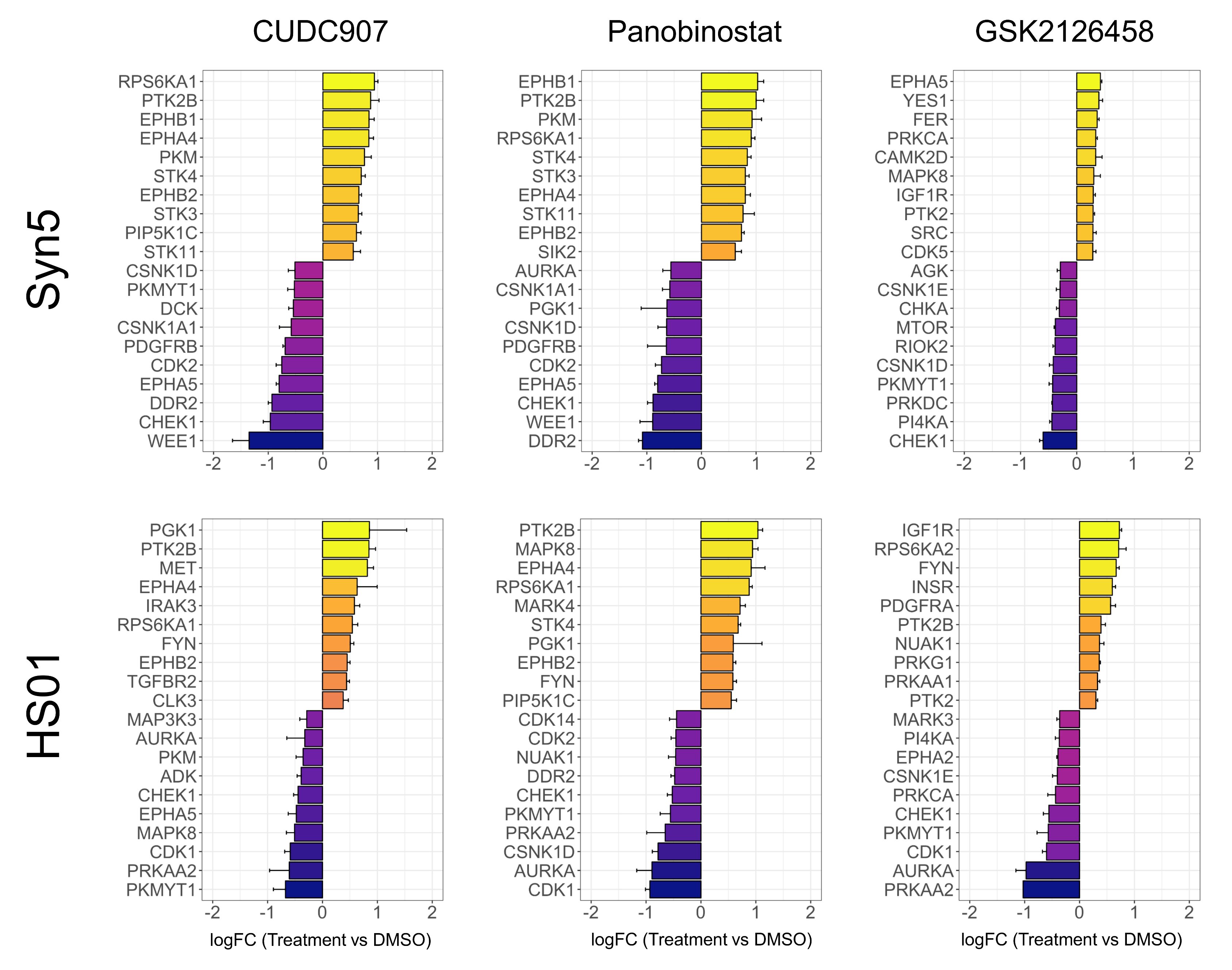

Supplement: S8 Fig — Top kinome changes in merlin deficient human AC (Syn5) and SC (HS01) treated with CUDC-907, Panobinostat or GSK2126458. Data presented are the median log2 fold change from 3 experiments, error bars are standard error. (TIF) [file pone.0197350.s017.tif]

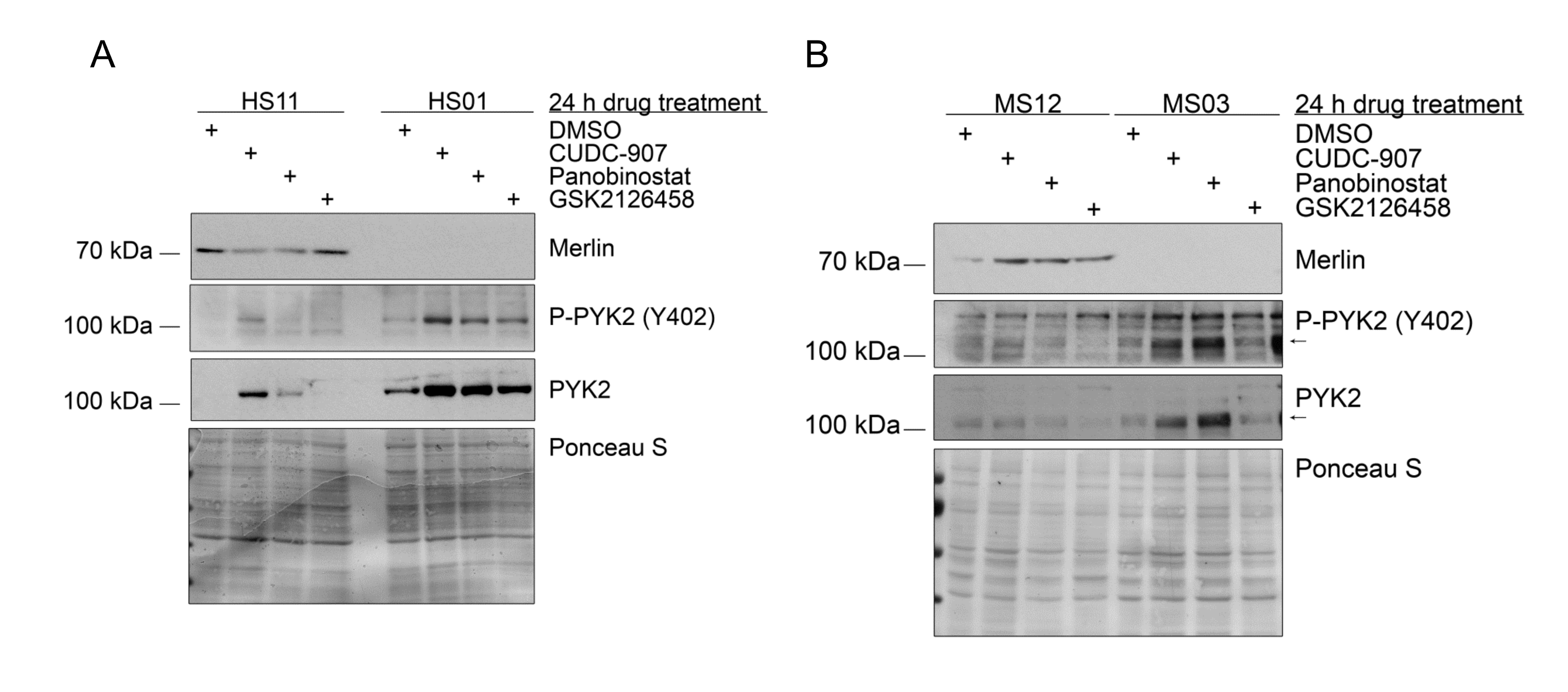

Supplement: S9 Fig — Both merlin-deficient mouse and human Schwann cells (SCs) increase PYK2 levels in response to DMSO, CUDC-907, Panobinostat and GSK2126458. Immunoblot analysis of human (HS11 and HS01) (A) and mouse (MS12 and MS03) (B) merlin-deficient SCs compared to wild-type control. Membranes were treated with merlin and PYK2 antibodies as indicated. 25 μg of lysates were used and equal loading was controlled by Ponceau S staining. These immunoblots are representative of three independent experiments. (TIF) [file pone.0197350.s018.tif]
