# Supplementary material for: Traditional and systems biology based drug discovery for the rare tumor syndrome neurofibromatosis type 2
Source: PLoS One. 2018 Jun 13;13(6):e0197350. doi: 10.1371/journal.pone.0197350 (PMC5999111; doi:10.1371/journal.pone.0197350)
Supplement: S1 Text — (DOCX) [file pone.0197350.s019.docx]

**S1 Text - *In vivo* testing of candidate compounds in a meningioma xenograft model – Immunohistochemical analysis**

Immunohistochemical analysis corroborated growth suppression in drug-treated meningioma xenografts. As HDAC inhibitors, both Panobinostat- and CUDC-907 treated tumors exhibited increased levels of acetylated proteins compared with those treated with the PI3K/mTOR inhibitor GSK2126458 or vehicle controls (Supplemental Figure 4A). Consistent with previous reports indicating that AKT is a downstream target of all three drugs, Western blot analysis showed decreased phospho-AKT (pAKT) at both the serine-473 and threonine-308 residues and its downstream signaling molecule pS6(S235/236) in drug-treated Ben-Men-1 cells (Supplemental Figure 4B). Note that GSK2126458 appeared to have a stronger inhibitory effect on p-AKTs and p-S6 than Panobinostat and CUDC-907. Similarly, we observed decreased pS6 in meningioma xenografts treated with these three drugs (Supplemental Figure 4C). We also detected fewer Ki67-positive cells in drug-treated tumors, compared with vehicle-treated tumors for all three compounds (Supplemental Figure 4D). Consistent with the kinome analysis (below), Ben-Men-1 tumors treated with any of these drugs showed increased pFAK(Y397) levels (Supplemental Figure 4E). pFAK expression is increased with treatment indicating drug induced conditions closer to wild-type merlin. Collectively, our results indicate that while GSK2126458, Panobinostat, and CUDC-907 all demonstrate some anti-tumor activity, Panobinostat and GSK2126458 had slightly higher activity than CUDC-907 in suppressing the growth of merlin-deficient meningioma *in vivo* under the conditions tested.
