# Supplementary material for: Traditional and systems biology based drug discovery for the rare tumor syndrome neurofibromatosis type 2
Source: PLoS One. 2018 Jun 13;13(6):e0197350. doi: 10.1371/journal.pone.0197350 (PMC5999111; doi:10.1371/journal.pone.0197350)
Supplement: S2 Text — (DOCX) [file pone.0197350.s020.docx]

## **S2 Text - Translation of *in vitro* to *in vivo* efficacy studies in meningioma and schwannoma models**

The studies reported above include systematic evaluation of a panel of 19 compounds selected for their potential activity against targets implicated in NF2 pathophysiology tested against a panel of human and murine, merlin-wildtype and -deficient cell lines derived from humans and mice. Importantly, the DRC analysis across histology, merlin status, and species indicated that tumor type showed the greatest effect on drug efficacy as measured by area under the curve (AUC), with cell line species having a moderate effect and merlin status having the least effect. Based on this analysis, the best DRC seen in both meningiomas and schwannoma cell lines, and analysis of agents most likely to be available for human trial, GSK2126458, Panobinostat, and CUDC-907 were selected to be evaluated *in vivo*.

I*n vivo*, there were notable differences between the meningioma xenograft model system (good tolerability and activity for all three drugs) and the GEM schwannoma model (high degree of toxicity with both GSK2126458 and CUDC-907 and tolerability, but no activity with Panobinostat). It is recognized that there are differences in cytochrome p450 enzymes within different strains of mice [1].  At least two of the drugs utilized (CUDC-907 and Panobinostat) are metabolized by cytochrome p450 enzymes that would impact GI and hepatic metabolism [2,3]. Differences in impact of the respective drugs between schwannomas and the meningiomas could be intrinsic to the basic biology of the respective tumors, the immunologic status (immune competent in schwannoma model vs immunocompromised in the meningioma model) or differences in development of the respective tumors in these experimental models.

References

1. Lofgren S, Hagbjork AL, Ekman S, Fransson-Steen R, Terelius Y. Metabolism of human cytochrome P450 marker substrates in mouse: a strain and gender comparison. Xenobiotica. 2004;34: 811-834.

2. Van Veggel M, Westerman E, Hamberg P. Clinical Pharmacokinetics and Pharmacodynamics of Panobinostat. Clin Pharmacokinet. 2017.

3. Cox J, Hein M, Luber C, Paron I, Nagaraj N, Mann M. MaxLFQ allows accurate proteome-wide label-free quantification by delayed normalization and maximal peptide ratio extraction Molecular & Cellular Proteomics. 2014.
