# Supplementary material for: Traditional and systems biology based drug discovery for the rare tumor syndrome neurofibromatosis type 2
Source: PLoS One. 2018 Jun 13;13(6):e0197350. doi: 10.1371/journal.pone.0197350 (PMC5999111; doi:10.1371/journal.pone.0197350)
Supplement: S3 Text — (DOCX) [file pone.0197350.s021.docx]

## **S3- Text - Transcriptome analysis of meningioma and schwannoma cell systems – Differential Gene Expression**

To directly examine the effects of merlin deficiency in human cells, we compared the isogenic AC pair Syn5/Syn1 and the isogenic SC pair HS01/HS11 (Figure 6B, 6C; Supplemental Tables 4 and 5). Complete merlin inactivation in Syn5, as evidenced by absence of merlin expression by Western blot (Supplemental Figure 1) and no representation in *NF2* mRNA of the targeted exon 8 (Supplemental Figure 5A), was associated with extensive changes to the transcriptome relative to the isogenic merlin-wildtype Syn1, with 1125 and 844 genes downregulated and upregulated, respectively at P <0.05 (Benjamini-Hochberg corrected). In human SCs, *NF2* suppression in HS01 to ~7% of normal HS11 mRNA levels resulted in fewer differentially expressed genes, with 561 and 546 significantly downregulated and upregulated, respectively. The mouse SC merlin-deficient MS03 compared to merlin-wildtype MS12 yielded 243 and 247 significantly downregulated and upregulated differentially expressed genes, respectively (Supplemental Tables 4 and 5B). Notably MS03 produced transcripts from *Nf2*, but, like Syn5, the targeted exon (in this case Cre targeting of the floxed exon 2) was completely absent (Supplemental Figure 5B). Mapping of the mouse differentially expressed genes to human orthologs was possible for 430 loci, but only 44 (10.2%) of these were also differentially expressed in the human SC, 22 concordant and 22 discordant for direction of change, a substantial difference consistent with the PCA analysis that cleanly distinguished the mouse and human SC cells by both of the first two principal components (Figure 6A).
