# Supplementary material for: Traditional and systems biology based drug discovery for the rare tumor syndrome neurofibromatosis type 2
Source: PLoS One. 2018 Jun 13;13(6):e0197350. doi: 10.1371/journal.pone.0197350 (PMC5999111; doi:10.1371/journal.pone.0197350)
Supplement: S4 Text — (DOCX) [file pone.0197350.s022.docx]

## **S4 Text - Transcriptome analysis of drug-treated arachnoidal/meningioma and Schwann cells**

The mouse SC showed comparatively less effect than the human SC with respect to the HDAC inhibitors, CUDC-907 and Panobinostat (Supplemental Tables 4 and 5B). In both Syn1 and Syn5 AC, the two HDAC inhibitors caused differential expression of more than 60% of the >17,000 genes reliably detected in each RNAseq assay. These differentially expressed genes were largely overlapping between the two drug treatments (Supplementary Figure 6B), suggesting that the HDAC inhibitor activity of CUDC-907 predominates over its proposed PI3K inhibitory activity. With both HDAC inhibitors, a substantial subset of genes showed a striking increase of >64 fold in expression (Supplemental Figure 6A) while treatment with GSK2126458 resulted in far fewer differentially expressed genes. This overall pattern was reproduced in the Syn6 established merlin-deficient meningioma line (Supplemental Tables 4 and 5A). Notably, a small proportion of the genes differentially expressed due to drug treatment changed in opposite directions in Syn1 and Syn5 revealing a potential effect of merlin presence/absence (Supplemental Table 9). Of the genes dysregulated by treatment with CUDC-907, 15 were altered in opposite directions and all but two of these were also differentially expressed in the baseline comparison of Syn5 cells to Syn1 cells. In the Panobinostat treatment 23 genes were differentially expressed in opposite directions in Syn1 and Syn5, and 7 of these were among the 15 CUDC-907 induced differences above. 18 of the 23 genes were also differentially expressed at baseline in the absence of drug treatment due to the difference in merlin status in Syn1 and Syn5. In contrast to the HDAC inhibitors, there were no genes that responded significantly in opposing directions to GSK2126458 treatment of Syn1 and Syn5.

In the human SC, Panobinostat treatment resulted in a greater number of differentially expressed genes than CUDC-907, and, while these differentially expressed genes again overlapped extensively, the correspondence was not as substantial as in the AC (Supplemental Figure 6B). A larger number of genes were differentially expressed due to GSK2126458 in the human SC lines and a greater number of these overlapped with effects of CUDC-907 not also seen with Panobinostat, suggesting that they may be associated with PI3 kinase inhibition rather than HDAC activity. There was also less variation in direction of response between merlin-wildtype and merlin-deficient SC than in the AC system, as only 5 genes from the Panobinostat treatment and 4 genes from the GSK2126458 treatment were differentially expressed in opposite directions in HS01 and HS11 (Supplemental Table 9). Detailed consideration of genes differentially expressed in opposite directions in merlin-wildtype versus merlin-deficient cells in response to these and future drug perturbations may prove informative in understanding the function of merlin within each cell type and in assessing the potential for combination therapies in which one compound enhances sensitivity to a second drug to selectively impact on NF2 tumors.
