# Supplementary material for: Traditional and systems biology based drug discovery for the rare tumor syndrome neurofibromatosis type 2
Source: PLoS One. 2018 Jun 13;13(6):e0197350. doi: 10.1371/journal.pone.0197350 (PMC5999111; doi:10.1371/journal.pone.0197350)
Supplement: S5 Text — (DOCX) [file pone.0197350.s023.docx]

# **S5 Text – Materials and Methods**

## **Inhibitors**

Axitinib, Selumetinib (AZD6244), Bortezomib (PS-341), Lapatinib (GW-572016) Ditosylate, Panobinostat (LBH589), Vorinostat (SAHA, MK0683), GDC-0941, Vismodegib (GDC-0449), OSU-03012 (AR-12), Everolimus (RAD001), Ganetespib (STA-9090), LY2157299, AR-42, GSK2126458 (GSK458), Trametinib (GSK1120212), GDC-0980 (RG7422), CUDC-907, AZD2014 and Perifosine (KRX-0401) were purchased from SelleckChem (Houston, TX). All inhibitors were prepared as 10 mM stock solutions in DMSO, except perifosine that was prepared in water. Stock solutions were aliquoted for single use size and stored at -80°C in glass vials. Semi-log serial dilutions in medium were prepared from the stock before each experiment keeping the DMSO concentration constant at 0.1%.

## **Cell lines and reagents**

To represent the biology of schwannoma formation, we employed an isogenic pair of primary fetal human SC lines, wild-type cells (HS11; ScienCell Lot#7228) and their counterpart in which merlin expression has been suppressed by *NF2*-shRNA treatment (HS01) [1][68]. Due to the difficulty of propagating adult primary human SC, a series of mouse SC lines was also screened (Table 1). These mouse SC lines included merlin-wildtype primary SC MS11 (from postnatal day 8 C57BL/6 mouse sciatic nerves (ScienCell Lot#7834) and MS12, isolated from sciatic nerves of adult *Nf2^flox2/flox2^* mice which spontaneously immortalized *in vitro*  [2] as well as the merlin-wildtype lines MS01, SC from the sciatic nerves of adult *Nf2^flox2/flox2^* mice[3][70] where *Nf2* exon 2 was deleted by *in vitro* infection with a Cre-expressing adenovirus (Ad-Cre)[4], MS02, a cell line established from a paraspinal schwannoma developed *in vivo* in an *Nf2^ex2-/-^* mouse generated by crossing *Nf2^flox2/flox2^* with a periostin-*Cre* [5], and MS03, *Nf2^ex2-/-^* SC generated from MS12 through *in vitro* by Ad-Cre infection.

The mouse cell lines MS01 and MS02 were cultured in 100 mm Corning CellBIND plates with growth medium containing DMEM/F12 (Life Technologies, Grand Island, NY) supplemented with N2 supplement (Life Technology, Burlington, ONT, Canada), 1% Penicillin/Streptomycin and maintained at 37ºC, 7% CO_2_. MS03 and MS12 were cultured in Corning BioCoat Poly-D-Lysine 100 mm culture dishes with DMEM:F12-HAM (1:1) (Sigma) plus N2 supplement (Life Technology), 50 μg/mL gentamicin (Life Technologies, Gibco), 2.5 μg/mL Fungizone (Life Technologies, Gibco), 2 μM forskolin (Calbiochem; Millipore) and 10 ng/mL Heregulin-b1 (Peprotech) growth medium. Primary mouse SC were cultured in 200 µg/mL poly-L-lysine (PLL) hydrobromide (Sigma-Aldrich) and 50 µg/mL laminin (Invitrogen) coated 100 mm plastic Corning culture dishes with growth medium containing 1:1 DMEM/F12 (Life Technologies, Grand Island, NY, USA) supplemented with N2 supplement (Life Technologies, Burlington, ONT, Canada), 2 µM forskolin, 10 ng/mL neuregulin, 5 ng/mL fibroblast growth factor and 1% Penicillin/Streptomycin and maintained at 37ºC, 7% CO_2_. The isogenic pair of human SC HS11 and HS01 were cultured in SC medium from ScienCell and maintained at 37ºC, 7% CO_2_. Mouse and human SC lines utilized in our studies were characterized/ authenticated by several methodologies including cell morphology, growth rates, marker immunostaining, western blot, and jumping gene analysis. All cell lines were tested for mycoplasma contamination with the LookOut mycoplasma PCR detection kit (Sigma-Aldrich). For Western blot analyses of kinase responses, we used MS12 wildtype an MS03 merlin-deficient mouse SC cultivated on 0.05 mg/ml Poly-L-Lysine/ 0.01mg/ml Laminine (Sigma Aldrich) coated plates with DMEM/F-12 medium (Sigma-Aldrich) mixed with N-2 Supplement (Thermo Fisher), Fungizone (Life Technologies) and Gentamycin (Thermo FisherScientific), 2 µM Forskolin (Merck Millipore) and 10 ng/ml human NRG1 (Peprotech).

For cell lines relevant to meningioma formation, we employed human lines including immortalized AC line clones that were modified using CRISPR/Cas9 genome editing (AC-CRISPR lines Syn1-5), immortalized merlin-deficient meningioma Ben-Men-1 (referred to as Syn6)[6], and patient-derived primary merlin*-*deficient MN lines (Syn7-12) lines including 3 WHO Grade I and 3 WHO Grade II (see Table 1 for details). Ben-Men-1 and AC-CRISPR cell lines were maintained under growth conditions described [6,7], and primary lines were established and maintained as reported[8].

## ***In vitro* screening pipeline**

For the meningioma-relevant cell panel, we established a primary chemical screening pipeline in which cells were seeded in a 384-well plate format and then assessed at 72h post-treatment using the CellTiter-Glo viability assay (Promega, Madison, WI). All assays were performed in triplicate, and all 19 compounds were assessed on one 384-well plate per cell line in order to avoid plate-to-plate variation. Controls included wells incubated with vehicle alone and medium alone. Detailed inhibitor treatment concentrations are described in the Results section. For transcriptome and kinome analyses, meningioma-relevant cells were treated with 100 nM of the chosen drugs for 24 h.For the schwannoma-relevant cell panel, the 7 cell lines were seeded into 384 well plates and incubated with the 19 compounds at half-log concentrations ranging from 0.001 µM to 10 µM in 0.1% DMSO for 48 hours. Cell viability was measured using the Cell-Titer Fluor assay (Promega). Each compound was tested in half or full plate columns (8 or 16 replicates). The DR assay was conducted twice for two mouse merlin-deficient SC lines (MS01, MS02) and the merlin-wildtype primary mouse SC (MS11). The two runs yielded good reproducibility; thus, the isogenic pairs of mouse and human SC lines were screened only once with all compounds as above. The negative control was 0.1% DMSO and the positive control was a toxic level of 50µM sirolimus or 27 µg/mL digitonin. Controls were run in 32 replicates and were used to calculate the plate Z-factor. The average Z factor for the eighty plates assayed was 0.7 indicating a good assay signal dynamic range and low variability[9].

For both cell types, dose-response measurements were normalized to vehicle control to calculate the percent viability for each well. Dose-response curves were then fit for each dataset using a 4/5-parameter logistic regression model that selected the best-fitting curve using a parameter optimization approach. IG10-IG90, Simpson area-under-the-curve and other efficacy metrics were then obtained from each curve using nplr. In order to assess the effect of covariates (drug used, tumor type, NF2 status, and organism) to the overall drug response, a multiple linear regression approach was used. This model estimates the individual effect of covariates on the drug response as measured by area under the curve[10]. All analyses including curve fitting, plotting, and multiple linear modeling were performed using R 3.2-3.4 and the R packages nplr, plotly, and ggplot2 [11-14].

The dosing for molecular studies was chosen as follows. For the meningioma-relevant cell lines, which included: an isogenic AC-CRISPR pair, Syn1 (merlin-wildtype) and Syn5 (merlin-deficient), and the immortalized MN line Syn6 (merlin-deficient), each of the three selected drugs was first tested to determine a concentration putatively affecting downstream signaling targets at 24h treatment time, without significant decrease in cell viability (decreased viability < 25%). Based on published reports [15-17]we observed dose responses (24h treatment) at 50 nM, 100 nM and 200 nM for the 3 selected compounds and chose to proceed at 100 nM for molecular assessments. For the schwannoma-relevant cell lines, the isogenic pair of human SC lines, HS11 (merlin-wildtype) and HS01 (merlin-suppressed) and a pair of spontaneously immortalized mouse isogenic SC cell lines, MS12 (merlin-wildtype) and MS03 (merlin-deficient) were selected. The dosing for each schwannoma-relevant cell line and each drug to perform the 24 h kinome and transcriptome studies was determined by performing dose response cell viability assays at 24h with the CellTiter-Fluor assay. We selected the concentration of the drug interpolated on the dose-response curve at 24 h that decreased 20-30% cell viability, while at 48 h produced a loss of viability equal or greater than the IG50. The human isogenic cell lines HS01/HS11 were treated with 0.03 µM CUDC-907, 0.2 µM Panobinostat, 0.3 µM GSK2126458. The mouse isogenic cell lines MS02/MS12 were treated with 0.02µM CUDC-907, 0.03 µM Panobinostat, 0.04 µM GSK2126458 and the mouse schwannoma cell line MS02 was treated with 0.08 µM CUDC-907, 0.12 µM Panobinostat, and 0.01 µM GSK2126458. Drugs were prepared as 10 mM Stock solutions in DMSO, aliquoted and stored at -80 °C. Drugs were diluted as indicated in medium from stock before each experiment (DMSO concentration was kept constant at 0.003 %). Treatment started at 80 % confluency, medium was refreshed before treatment. The cells were incubated with each drug for 24 h.

## **Immunostaining**

Cells were seeded on 200 µg/mL poly-L-lysine (PLL, Sigma-Aldrich) coated German glass coverslips (Carolina Biological). Cells were fixed in 4% paraformaldehyde, permeabilized, and immunostained as previously described[4]. Primary antibodies: S100 (1:200; Dako Cytomation), Human Nuclear Antigen (1:300; Millipore MAB1281). Alexa Fluor633-conjugated phalloidin (1:200), DAPI (1:200). Fluorescence Images were acquired with a Zeiss LSM710 Confocal microscope and ZEN software.

## **Compound efficacy in a quantifiable, orthotopic merlin-deficient meningioma model**

This animal study was approved by the Institutional Animal Care and Use Committee at Nationwide Children’s Hospital. Actively-growing, luciferase-expressing Ben-Men-1-LucB cells [18] were washed and resuspended in phosphate buffered saline (PBS). Approximately 1x10^6^ cells were stereotactically injected into the skull base of eight-to-12 week-old severe combined immunodeficiency mice (SCID-C.B17, Taconic) 1.5 mm anterior and 1.5 mm to the right of the bregma and 4.5 mm below the skull surface as previously described [18]. To assess tumor engraftment, bioluminescence imaging (BLI) was performed biweekly on injected mice using a Xenogen IVIS^®^ Spectrum imaging system (Caliper). Following successful tumor engraftment, defined as detectable and increasing bioluminescence signals over at least two timepoints, tumor-bearing mice were randomized into two groups (n = 8 each) and treated with a drug or the corresponding vehicle in which the drug was formulated. GSK2126458 was formulated in 0.5% (hydroxypropyl)methyl cellulose and 0.1% Tween-80 and delivered to mice at a dose of 2 mg/kg by oral gavage once daily [19]. Panobinostat was first dissolved in dimethyl sulfoxide (DMSO), then diluted 50-fold in 5% dextrose in water, and administered to animals at a dose of 20 mg/kg via intra-peritoneal injection every other day [20]. CUDC-907 was solubilized in either propylene glycol or 30% Captisol^®^ and administered at a dose of 25 mg/kg by oral gavage once daily [17]. These three drugs were evaluated with independent groups of mice, and the effects of treatment were monitored by BLI [18].

## **Immunohistochemistry**

Following euthanasia, the heads of drug- or vehicle-treated mice were dissected, fixed in 10% phosphate-buffered formalin, and then processed for decalcification[18]. Decalcified heads were incubated in 10% sucrose overnight and embedded in paraffin, followed by tissue sectioning. Thin (5-µm) sections were deparaffinized and stained with hematoxylin and eosin. For immunostaining, sections were treated overnight with a primary antibody against acetylated lysine (#9441, Cell Signaling), p-S6(S235/236) (#4858, Cell Signaling), Ki67 (RM-9106-S, Neomarkers), or p-FAK(Y397) (#700255, Invitrogen). After washing, stained sections were incubated with an HRP-conjugated secondary antibody, followed by color development with AEC chromogen (ScyTek) and counterstaining with hematoxylin. Negative controls were treated with the same procedure but without the primary antibody.

## **Western blot analysis**

Western blotting was performed as previously described[18,21]. Briefly, treated cells were lysed and equal amounts of protein lysates were resolved by SDS-polyacrylamide gel electrophoresis, followed by protein transfer to a PVDF membrane. Primary antibodies used included the antibody against pAKT(S473) (#4060, Cell Signaling), pAKT(T308) (#2965, Cell Signaling), merlin (#12888, Cell Signaling), PYK2 (ab32571, Abcam), pPYK2(Y402) (#3291, Cell Signaling), pS6(S235/236) (#4858, Cell Signaling), pERKs (#4370, Cell Signaling), pp90RSK(S380) (#9341 and 11989, Cell Signaling), and GAPDH (#5174, Cell Signaling or sc-32233 Santa Cruz). For detection of merlin in human AC-CRISPR and meningioma cell lines, anti-merlin polyclonal antibodies N21 and C26, were used, which have been described[22].

## **RNA isolation**

For transcriptome analyses, RNA was isolated from all meningioma-relevant and mouse SC lines using TRIzol reagent (Ambion/Life Technologies; Grand Island, NY) according to the manufacturer’s instructions. Pelleted cells were resuspended TRIzol reagent using micro-dounce homogenization then extracted with chloroform, followed by isopropanol precipitation of RNA from the aqueous phase and an 80% ethanol wash. RNA pellets were solubilized in 30-50 µl of RNase-free water (Ambion, AM9937). For HS01 and HS11 SC, RNA was prepared from flash-frozen cell pellets with a Qiagen RNeasy Plus Mini Kit. RNA quality was assessed using the Agilent Bioanalyzer Tapestation 2200 (Agilent Technologies, Santa Clara CA).

## **Transcriptome analysis**

For all cell lines other than HS01 and HS11, mRNA libraries for RNAseq were made using TruSeq Stranded mRNA Library Preparation Kits from Illumina RS-122-2101 and RS-122-2102 – with single 6n indexes and RS-122-2103 – with dual 8n indexes. Libraries were analyzed using D1000 tape of Agilent Tapestation 2200 and by quantitative qPCR with master mix and standards from CAPA Biochemicals, using LightCycler480 96-well. These libraries were multiplexed, pooled and sequenced on multiple lanes of Illumina HiSeq2000 and 2500 platforms in multiple runs, generating paired-end sequencing reads of 51-76 bp. Quality checking of sequence reads was assessed by fastQC (v.0.10.1) (<http://www.bioinformatics.babraham.ac.uk/projects/fastqc/>) and if necessary, sequences were processed to trim bases with exceptionally low quality scores using fastq_quality_filter from FASTX Tool Kit (v.0.0.13) (http://hannonlab.cshl.edu/fastx_toolkit/), Sickle (v.1.2)[23]), Cutadapt (v.1.9.1)[24] and custom Perl scripts. For HS01 and HS11, RNA was quantitated by Qubit assay and 4 ug RNA was used as input for library preparation using a Kapa Stranded mRNA-seq Kit according to manufacturer’s protocol, using TruSeq adapters for indexing and library amplification of 10 cycles. Libraries concentration was determined by Qubit and quality assessment and average fragment length determined by Agilent TapeStation. Equimolar 8- or 9-plex pools were run on a NextSeq 500 using a v2 high output 75 cycle kit. All samples were from experiments performed in triplicate except HS01-HS11 baseline DMSO treatment which was run in quadruplicate (except one HS01 run failed QC thresholds and was subsequently discarded).

Sequence reads from human and mouse samples were aligned to human reference genome Ensembl GRCh37 (v.71) and mouse reference genome GRCm38 (v.75) respectively, using GSNAP (v. 12-19-2014) with options –N 1 –B 3 --quality-unk-mismatch=1[25]. Quality checking of alignments was assessed by a custom script utilizing Picard Tools (<http://broadinstitute.github.io/picard/>), RNASeQC[26], RSeQC[27] and samTools[28]. Gene level counts were tabulated using BedTools’s multibamcov algorithm (v. 2.17.0)[29] on unique alignments, which were identified by “NH:i:1” tag, for each library based on Ensembl gene annotation for human (GRCh37 v.71) or mouse (GRCm38 v.75). Differentially expressed genes in pair-wise comparisons were identified by edgeR’s quasi-likelihood F test (v. 3.12)[30], which was run at the R platform (v. 3.2.2) on genes with greater than 20 counts across replicates per condition in pair-wise comparisons after filtering out short genes with length < 250 nt and rRNA and tRNA genes. Gene ontology enrichment analysis for each comparison was performed on differentially expressed genes at Benjamini Hochberg (BH)[31] adjusted p values < 0.05, where all the analyzed genes in a given comparison was used as a background list, using DAVID (v. 6.8)[32]. Drug-gene interaction data for differentially expressed genes with BH adjusted p value < 0.05 was retrieved from DGIdb[33]. Principal components analysis was performed on baseline samples rank-normalized counts averaged across replicates to assess similarity between different cell lines used in this study.

## **Lysis and MIB chromatography**

Broad spectrum Type I kinase inhibitors (CTx-0294885, VI-16832, PP58, Purvalanol B, UNC-21474, and UNC-8088A), custom-synthesized with hydrocarbon linkers and terminal amine groups were covalently attached to ECH-activated Sepharose beads as previously described[34]. Cells were rinsed in PBS and processed in lysis buffer (50 mM HEPES, 150 mM NaCl, 0.5% Triton X-100, 1 mM EDTA, 1 mM EGTA, at pH 7.5 containing 10 mM NaF, 2.5 mM NaVO4, cOmplete protease Inhibitor Cocktail (Roche), and 1% Phosphatase Inhibitor Cocktails 2 and 3 (Sigma). 2-5mg total protein lysate was gravity-flowed over a mixture of the six kinase inhibitor-linked beads (175uL total beads), followed by 30 volumes of washes with high salt (1M NaCl) and low salt (150mM NaCl) lysis buffer, then 500uL of low salt lysis buffer containing 0.1% SDS. Bound proteins were eluted by boiling with 0.5% SDS and 1% β-mercaptoethanol in 100mM Tris-HCl, pH 6.8, 2X 15 minutes, treated with DTT (5mM, 25min at 60°C) and Iodoacetamide (20mM, 30min in the dark at RT), and spin-concentrated to 100μL (Amicon Millipore Amicon Ultra-4, 10K cutoff) before Methanol/Chloroform precipitation. Proteins were trypsinized overnight at 37°C and drug-treated or DMSO samples labeled with TMT sixplex reagents (Thermo) according to manufacturer instructions. Baseline kinome samples were not labeled. Samples were dried down in a speed-vac and cleaned with C-18 spin columns (Pierce).

## **Mass Spectrometry and Analysis**

For TMT-labeled samples, 5% of each sample was first run on a 60 min LC gradient and then equalized on total peptide content before combining. Peptides were resuspended in 2% ACN and 0.1% Formic Acid. 30% of the final peptide suspension was injected onto an Easy nLC-1000 through a Thermo Easy-Spray 75μm x 25cm C-18 column and separated on a 300min gradient (5-40% ACN). ESI parameters: 3e6 AGC MS1, 80ms MS1 max inject time, 1e5 AGC MS2, 100ms MS2 max inject time, 20 loop count, 1.8 m/z isolation window, 45s dynamic exclusion. Spectra from TMT-labeled runs were searched against the Uniprot/Swiss-Prot database with Sequest HT on Proteome Discoverer software. Label-free runs for baseline kinome were searched against the Uniprot/Swiss-Prot database using MaxQuantLFQ[15] and default search parameters (except only unique peptides were used and match between runs was enabled). LFQ intensities were used for comparison. For TMT-labeled samples, only peptides with medium or greater confidence (5% FDR) were considered for quantitation, and peptides with greater than 75% coisolation interference were omitted. Filtered peptide level data from all the replicates for each condition were pooled together for analysis. Data for each treated sample was processed as a fold change (log2) relative to DMSO-treated control and was plotted as mean +/- standard error of the peptide ratio for each kinase. Differentially expressed kinases in pair-wise comparisons were identified by using a non-parametric Wilcoxon rank sum test. The significant kinases were selected after FDR correction using adjusted p values < 0.05. Heat maps were generated using GENE-E software (BROAD institute) or the R package pheatmap and colored using the R package viridis. Kinome trees were generated using Kinome Render (<http://bcb.med.usherbrooke.ca/kinomerender.php)>[35].

## **Integrative Analysis**

To compare the transcriptome and kinome experiments, we identified the intersecting kinases in the two datasets. For the baseline kinome experiment, the two genotypes (merlin*-*wildtype and merlin-wildtype) were measured independently. Therefore, the kinome and transcriptome log2 fold changes could be directly compared. The log2 fold changes of the merlin-deficient and merlin-wildtype kinome were plotted against the log2 fold changes of the merlin-deficient and merlin-wildtype transcriptome (R package ggplot2). Kinases with a fold change greater than 1 or less than -1 in the transcriptome and a fold change greater than 0.5 or less than -0.5 in the kinome were labeled using the color scheme described in the figure schematic legend (R package ggrepel) [36].

For the post-treatment kinome analysis, the MIB/MS experiment was performed using labeled peptides and yielded relative intensities of the treated samples in comparison to the vehicle control. Therefore, in the kinome dataset, a log2 fold change across two treated cell lines (merlin-deficient vs merlin-wildtype) accounts for the baseline difference between the merlin-deficient and -wildtype cell lines, and examines genotype-dependent and treatment-specific changes to the kinome. However, in the transcriptome, the comparison of drug treated merlin-deficient cells with merlin-wildtype cells does not account for the baseline differences between cell lines. In order to compare kinome to transcriptome data, we normalized the transcriptome data using the following strategy: we obtained counts per million for all genes in all samples using the cpm() function in the R package edgeR. For each drug and technical replicate, we generated ratios of these counts by dividing drug-treated counts by averaged vehicle-treated gene counts. Then, the mean drug/vehicle gene count ratio across all merlin-deficient replicates for a given drug was normalized by the mean drug/vehicle gene count ratio across all merlin-wildtype replicates for the same drug to generate a log2 fold change that accounts for baseline differences that can be attributed to the *NF2* genotype (merlin-deficient/merlin-wildtype ratio). These transcriptome ratios were then plotted against the merlin-deficient/merlin-wildtype kinome log2 fold change data for the same drug using the R package ggplot2. Kinases with a fold change greater than 1 or less than -1 in the transcriptome datasets and a fold change greater than 0.5 or less than -0.5 in the kinome datasets were labeled using the color scheme described in the figure legend (R package ggrepel).

References

1. Petrilli AM, Garcia J, Bott M, Klingeman Plati S, Dinh CT, Bracho OR, et al. Ponatinib promotes a G1 cell-cycle arrest of merlin/NF2-deficient human schwann cells. Oncotarget. 2017;8: 31666-31681.

2. Manent J, Oguievetskaia K, Bayer J, Ratner N, Giovannini M. Magnetic cell sorting for enriching Schwann cells from adult mouse peripheral nerves. J Neurosci Methods. 2003;123: 167-173.

3. Giovannini M, Robanus-Maandag E, van der Valk M, Niwa-Kawakita M, Abramowski V, Goutebroze L, et al. Conditional biallelic Nf2 mutation in the mouse promotes manifestations of human neurofibromatosis type 2. Genes Dev. 2000;14: 1617-1630.

4. Petrilli AM, Fuse MA, Donnan MS, Bott M, Sparrow NA, Tondera D, et al. A chemical biology approach identified PI3K as a potential therapeutic target for neurofibromatosis type 2. Am J Transl Res. 2014;6: 471-493.

5. Gehlhausen JR, Park SJ, Hickox AE, Shew M, Staser K, Rhodes SD, et al. A murine model of neurofibromatosis type 2 that accurately phenocopies human schwannoma formation. Hum Mol Genet. 2015;24: 1-8.

6. Puttmann S, Senner V, Braune S, Hillmann B, Exeler R, Rickert CH, et al. Establishment of a benign meningioma cell line by hTERT-mediated immortalization. Lab Invest. 2005;85: 1163-1171.

7. James MF, Han S, Polizzano C, Plotkin SR, Manning BD, Stemmer-Rachamimov AO, et al. NF2/merlin is a novel negative regulator of mTOR complex 1, and activation of mTORC1 is associated with meningioma and schwannoma growth. Mol Cell Biol. 2009;29: 4250-4261.

8. James MF, Lelke JM, Maccollin M, Plotkin SR, Stemmer-Rachamimov AO, Ramesh V, et al. Modeling NF2 with human arachnoidal and meningioma cell culture systems: NF2 silencing reflects the benign character of tumor growth. Neurobiol Dis. 2008;29: 278-292.

9. Zhang JH, Chung TD, Oldenburg KR. A Simple Statistical Parameter for Use in Evaluation and Validation of High Throughput Screening Assays. J Biomol Screen. 1999;4: 67-73.

10. Faraway JJ. Linear Models with R. Second Edition ed. Boca Raton, FL: Chapman & Hall/CRC Press; 2009.

11. R Core Team. R: A Language and Environment for Statistical Computing. . 2017.

12. Sievert C, Parmer C, Hocking T, Chamberlain S, Ram K, Corvellec M, et al. plotly: Create Interactive Web Graphics via 'plotly.js'. . 2017.

13. Commo F, Bot BM. nplr: N-Parameter Logistic Regression. . 2016.

14. Wickham H. ggplot2: Elegant Graphics for Data Analysis. New York: Springer-Verlag; 2016.

15. Song X, Wang J, Zheng T, Song R, Liang Y, Bhatta N, et al. LBH589 Inhibits proliferation and metastasis of hepatocellular carcinoma via inhibition of gankyrin/STAT3/Akt pathway. Mol Cancer. 2013;12: 114-4598-12-114.

16. Leung E, Kim JE, Rewcastle GW, Finlay GJ, Baguley BC. Comparison of the effects of the PI3K/mTOR inhibitors NVP-BEZ235 and GSK2126458 on tamoxifen-resistant breast cancer cells. Cancer Biol Ther. 2011;11: 938-946.

17. Qian C, Lai CJ, Bao R, Wang DG, Wang J, Xu GX, et al. Cancer network disruption by a single molecule inhibitor targeting both histone deacetylase activity and phosphatidylinositol 3-kinase signaling. Clin Cancer Res. 2012;18: 4104-4113.

18. Burns SS, Akhmametyeva EM, Oblinger JL, Bush ML, Huang J, Senner V, et al. Histone deacetylase inhibitor AR-42 differentially affects cell-cycle transit in meningeal and meningioma cells, potently inhibiting NF2-deficient meningioma growth. Cancer Res. 2013;73: 792-803.

19. Knight SD, Adams ND, Burgess JL, Chaudhari AM, Darcy MG, Donatelli CA, et al. Discovery of GSK2126458, a Highly Potent Inhibitor of PI3K and the Mammalian Target of Rapamycin. ACS Med Chem Lett. 2010;1: 39-43.

20. Qian DZ, Kato Y, Shabbeer S, Wei Y, Verheul HM, Salumbides B, et al. Targeting tumor angiogenesis with histone deacetylase inhibitors: the hydroxamic acid derivative LBH589. Clin Cancer Res. 2006;12: 634-642.

21. Schulz A, Buttner R, Hagel C, Baader SL, Kluwe L, Salamon J, et al. The importance of nerve microenvironment for schwannoma development. Acta Neuropathol. 2016;132: 289-307.

22. Wiederhold T, Lee MF, James M, Neujahr R, Smith N, Murthy A, et al. Magicin, a novel cytoskeletal protein associates with the NF2 tumor suppressor merlin and Grb2. Oncogene. 2004;23: 8815-8825.

23. Joshi NA FJ. Sickle: A sliding-window, adaptive, quality-based trimming tool for FastQ files. . 2011;(Version 1.33) [Software].

24. MARTIN M. Cutadapt removes adapter sequences from high-throughput sequencing reads. EMBnet journal. 2011;17: 10-12.

25. Wu TD, Nacu S. Fast and SNP-tolerant detection of complex variants and splicing in short reads. Bioinformatics. 2010;26: 873-881.

26. DeLuca DS, Levin JZ, Sivachenko A, Fennell T, Nazaire MD, Williams C, et al. RNA-SeQC: RNA-seq metrics for quality control and process optimization. Bioinformatics. 2012;28: 1530-1532.

27. Wang L, Wang S, Li W. RSeQC: quality control of RNA-seq experiments. Bioinformatics. 2012;28: 2184-2185.

28. Li H, Handsaker B, Wysoker A, Fennell T, Ruan J, Homer N, et al. The Sequence Alignment/Map format and SAMtools. Bioinformatics. 2009;25: 2078-2079.

29. Quinlan AR, Hall IM. BEDTools: a flexible suite of utilities for comparing genomic features. Bioinformatics. 2010;26: 841-842.

30. Robinson MD, McCarthy DJ, Smyth GK. edgeR: a Bioconductor package for differential expression analysis of digital gene expression data. Bioinformatics. 2010;26: 139-140.

31. Benjamini Y. HY. Controlling the False Discovery Rate: A Practical and Powerful Approach to Multiple Testing.
 . Journal of the Royal Statistical Society Series B (Methodological). 1995;57: 289-300.

32. Davis MI, Hunt JP, Herrgard S, Ciceri P, Wodicka LM, Pallares G, et al. Comprehensive analysis of kinase inhibitor selectivity. Nat Biotechnol. 2011;29: 1046-1051.

33. Griffith M, Griffith OL, Coffman AC, Weible JV, McMichael JF, Spies NC, et al. DGIdb: mining the druggable genome. Nat Methods. 2013;10: 1209-1210.

34. Lang B, Zhang L, Jiang G, Hu L, Lan W, Zhao L, et al. Control of cortex development by ULK4, a rare risk gene for mental disorders including schizophrenia. Sci Rep. 2016;6: 31126.

35. Chartier M, Chenard T, Barker J, Najmanovich R. Kinome Render: a stand-alone and web-accessible tool to annotate the human protein kinome tree. PeerJ. 2013;1: e126.

36. Slowikowski, K. ggrepel: Repulsive Text and Label Geoms for 'ggplot2'. R package version 2016;0.6.5.
